# Investigating Climate Compatible Development Outcomes and their Implications for Distributive Justice: Evidence from Malawi

**DOI:** 10.1007/s00267-017-0890-8

**Published:** 2017-05-24

**Authors:** Benjamin T. Wood, Claire H. Quinn, Lindsay C. Stringer, Andrew J. Dougill

**Affiliations:** Sustainability Research Institute, School of Earth and Environment, University of Leeds, Leeds, LS2 9JT UK

**Keywords:** Climate change, development; equity, social justice, adaptation, mitigation

## Abstract

Governments and donors are investing in climate compatible development in order to reduce climate and development vulnerabilities. However, the rate at which climate compatible development is being operationalised has outpaced academic enquiry into the concept. Interventions aiming to achieve climate compatible development “wins” (for development, mitigation, adaptation) can also create negative side-effects. Moreover, benefits and negative side-effects may differ across time and space and have diverse consequences for individuals and groups. Assessments of the full range of outcomes created by climate compatible development projects and their implications for distributive justice are scarce. This article develops a framework using a systematic literature review that enables holistic climate compatible development outcome evaluation over seven parameters identified. Thereafter, we explore the outcomes of two donor-funded projects that pursue climate compatible development triple-wins in Malawi using this framework. Household surveys, semi-structured interviews and documentary material are analysed. Results reveal that uneven outcomes are experienced between stakeholder groups and change over time. Although climate compatible development triple-wins can be achieved through projects, they do not represent the full range of outcomes. Ecosystem—and community-based activities are becoming popularised as approaches for achieving climate compatible development goals. However, findings suggest that a strengthened evidence base is required to ensure that these approaches are able to meet climate compatible development goals and further distributive justice.

## Introduction

**Fig. 1:**
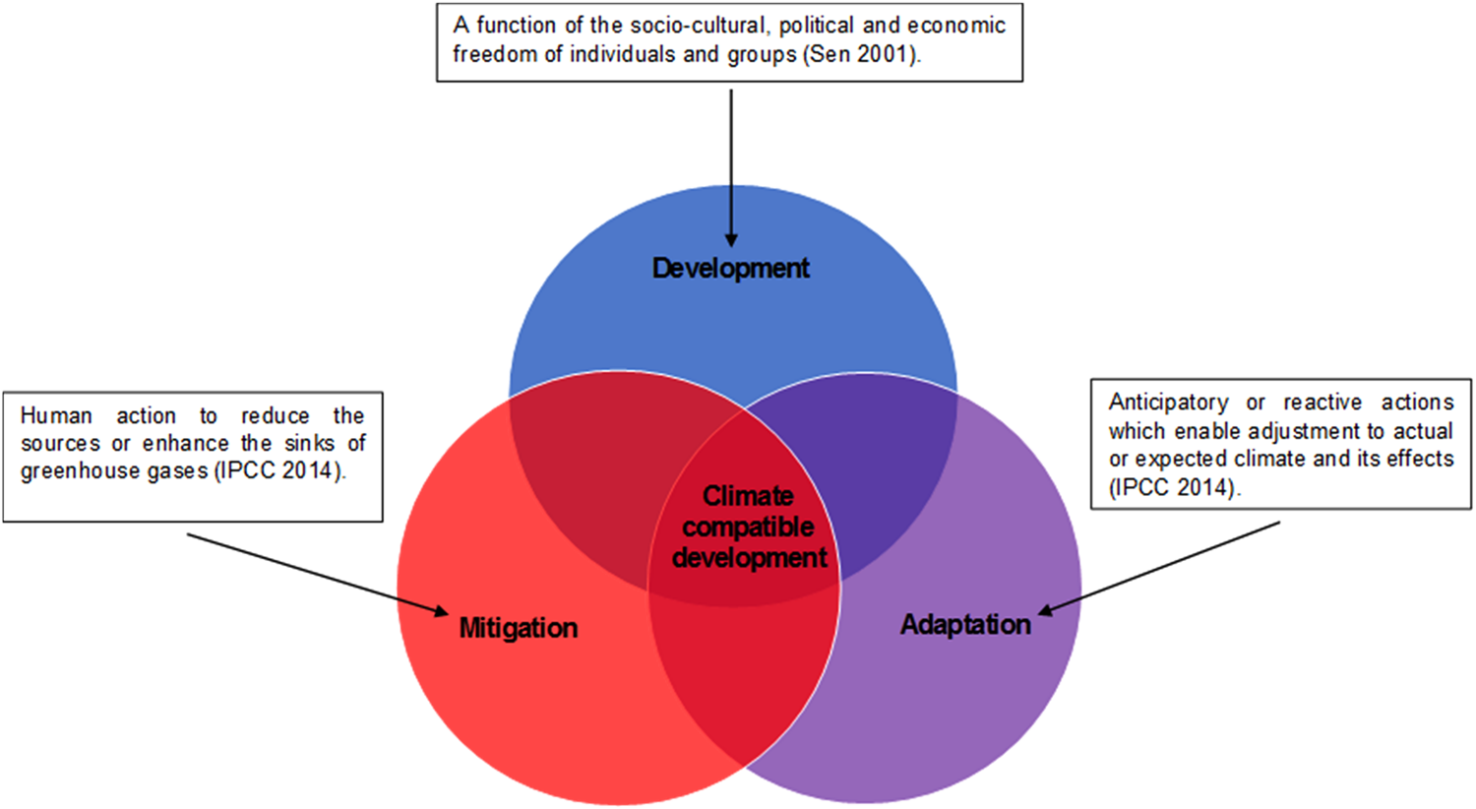
The popularised depiction of CCD and definitions of its components (Adapted from: Mitchell and Maxwell 2010; Sen 2001; IPCC 2014)

Climate compatible development (CCD) is defined as “development that minimises the harm caused by climate impacts, while maximising the many human development opportunities presented by a low emissions, more resilient future” (Mitchell and Maxwell 2010: 1). It is used as a framework for guiding policies, programmes and projects towards “triple-wins” across development, mitigation and adaptation; seeking to enhance synergies and areas of overlap between each component as well as reducing conflict amongst them (Fig. 1).

Climate change and development present complex policy problems (Hulme 2011). By offering simultaneous solutions to both, CCD has gained traction amongst academics and policymakers (Nunan 2017). Governments and donors are currently investing in CCD to reduce vulnerabilities (Stringer et al. 2014), where vulnerability is a function of: exposure to socio-economic, political and environmental (including climatic) shocks; sensitivity to these shocks and stressors; and capacities to adapt to them (Gaillard 2010).

Despite such investment, the CCD literature remains in its infancy. Research has empirically examined circumstances in which CCD wins might be achievable (e.g., Rahn et al. 2014; Bryan et al. 2013). A growing literature is also assessing the development co-benefits (anticipated or unanticipated positive impacts) of mitigation and adaptation (e.g., West et al. 2013). Other work has assessed drivers of, and challenges for, CCD (Ellis et al. 2013); appraised its potential for guiding policymaking and natural resource valuation (Huxham et al. 2015); and presented lessons for its operationalisation (Dyer et al. 2013; Broto et al. 2015).

Overall, research has focussed on facilitating and showcasing how CCD wins can be achieved and, to a lesser extent, identifying the winners. However, CCD interventions stand to create multi-level patterns of both benefits and negative side-effects (NSE) that may differ across time and space and have diverse consequences for individuals and groups (“winners” and “losers”) (Tompkins et al. 2013). Outcomes created by development and, to a lesser extent, adaptation interventions, have been rigorously examined (e.g., McDermott and Schreckenberg 2009; Osbahr et al. 2010). Yet, linked to a shortage of suitable evaluation tools, analyses that consider the full range of CCD outcomes are scarce, meaning that the literature often presents only a partial view.

Framing CCD research towards wins and winners can encourage policy and practice that is overly optimistic about what CCD can achieve and/or lacks safeguards to prevent or cushion NSEs. Research that recognises the full range of CCD outcomes is urgently required to ensure CCD investments are both effective (successfully achieving development, mitigation and adaptation benefits) and efficient (achieving benefits without incurring associated NSEs). It would also reveal CCD’s distributive justice implications, i.e., whether and how it creates or exacerbates economic (un)freedoms that determine whether people can pursue ends that they value (Sen 2001). Understanding these implications is crucial because CCD professes to be a “development first” approach (Picot and Moss 2014). Nevertheless, the CCD literature has paid limited attention to distributive justice.

Multiple identities, global inequalities and diverse cross-scale experiences with climate impacts and policy outcomes make a universal standard of distributive justice impossible to define with regard to CCD (Fisher 2015). Rather, distributive justice is circumstantial and must be “negotiated and generated in the context of conflicting views and interests” (Paavola and Adger 2006: 600–601). This requires that individuals and groups who are impacted by CCD are afforded procedural justice: they must be granted recognition, or equality of status, and participatory opportunities within decision-making processes (Sen 2001).

Predominant theories of distributive justice are underpinned by universal laws, which is problematic because they overlook how different contexts shape empirical justice claims (Walzer 1983). Liu (2010) argues that theories can be grouped into one of four main “types”: (i) utilitarianism, which prioritises aggregate welfare maximisation; (ii) egalitarianism, which prioritises the reduction of societal inequality; (iii) libertarianism, which prioritises individual freedom; and (iv) contractarianism, which requires that the least privileged in society should be made as well-off as possible. Terms including “equity” and “fairness” are used interchangeably with distributive justice within the literature (McDermott et al. 2012), with each implying “fair treatment and due reward” (Schroeder and Pisupati 2010: 13). The lineage of these terms is drawn upon in this article.

Each type of distributive justice theory has gained traction within climate and development research. The literature often recourses to utilitarian assumptions, considering climate impacts and development progress to “matter” only when they impact on well-being and can be quantified monetarily (Adger et al. 2011). Egalitarian thinking permeates policy and action that emphasises the reduction of global inequalities (e.g., the Sustainable Development Goals) and, in regards to climate change, equal entitlements to the atmosphere and equal rights to be protected from climate impacts (see Klinsky and Dowlatabadi 2009). Efforts emphasising protection from climate impacts caused by others (Ibid.) also show libertarian thinking, while contractarian thinking dominates in calls to protect the most vulnerable from climate and development shocks and stresses (Gaillard 2010).

This article seeks to address research gaps concerning CCD outcomes and distributive justice. We analyse patterns of multi-level, cross-scale benefits and NSEs resulting from implementation of two donor-funded projects that pursued CCD triple-wins in Malawi. Together, these projects formed the *Enhancing Community Resilience Programme* (ECRP), which sought to improve the lives of over 600,000 Malawians whose predominantly agriculture-based livelihoods are acutely threatened by regular and worsening climate shocks and stresses (including dry spells and drought, heavy rains and flooding, windstorms). The projects professed to target particularly vulnerable households (e.g., the extremely resource-poor, elderly-headed, female-headed, those with disabled or chronically ill adult members) whose capacity to adapt to climate shocks and stresses is extremely low. This represents a contractarian distributive justice approach.

The projects drew on a range of community—and ecosystem-based activities to pursue CCD goals. Community-based project theory stresses the need to involve “communities” made up of groups of local people bound together by considerations such as culture, identity and place, in different stages of project implementation (Ayers and Forsyth 2009). Ecosystem-based activities pursue benefits by drawing on natural resources and the services they provide (Reid 2015). Adopted together, they are considered to create CCD outcomes in a more cost-effective, flexible and less path-dependent way than “top-down” solutions which are implemented without local involvement (Mansuri and Rao 2004).

## Research Design and Methodology

### Research Context and Case Study Approach

Malawi is one of the world’s most climate vulnerable countries (Barrett 2013). It faces multiple interrelated social, political, economic and environmental stressors, ranking 173rd out of 187 countries assessed by the Human Development Index (UNDP 2015), and its population faces various forms of deprivation (OPHI 2013). High levels of financial and resource poverty; food insecurity; population growth; limited access to safe water and hygiene; a high prevalence of HIV/AIDS; low literacy rates, poor access to clean, modern energy supply; and a limited coverage of transport and communications infrastructure across the country, present persistent challenges. Malawi is also highly aid dependent, with international support accounting for approximately 37 per cent of government spending and severe budgetary constraints restricting investment in public services and social protection (AidData 2016).

Dominant economic sectors (notably agriculture) and associated livelihoods are highly sensitive to climate change impacts (GoM 2006). People in the country already contend with extreme weather events, such as droughts and floods. Future climate projections suggest a high probability that extreme weather events in Malawi will increase and worsen throughout the 21st century but climate information is poorly integrated into national level policymaking (Vincent et al. 2017). Due to wider development problems, adaptive capacity is also low.

Projects pursuing CCD goals are increasingly being implemented across the country. ECRP comprised two projects: (1) the *Developing Innovative Solutions with Communities to Overcome Vulnerability with Enhanced Resilience* project (DISCOVER), and: (2) the *Enhancing Community Resilience Project* (ECRProject). ECRP projects were chosen for study because they have the most wide-reaching distributive justice implications of all CCD projects we identified as being implemented in Malawi (Online Appendix A).

The projects were financed by the UK, Norwegian and Irish Governments and were predominantly designed to help households overcome development challenges, with particular emphasis on improving food and nutrition security; increasing income and asset ownership; lessening dependence on unclean inefficient forms of energy; and reducing environmental resource degradation (DFID No Date). They also aimed to help households in Malawi adapt to climate impacts (Ibid.). In addition, the projects sought to contribute to carbon savings, therefore incorporating mitigation. In programmatic literature, projects were discussed in terms of their propensities to achieve CCD (Ibid.).

DISCOVER and the ECRProject provided direct support to 305,000 and 298,500 local people, respectively, with a range of ecosystem—and community-based activities: conservation agriculture (CA), small-scale irrigation, livestock production, solar lighting, improved cookstoves, post-harvest management, seed multiplication schemes, forestry activities and village savings and loans associations (VSLAs). Both projects began in September 2011 and ran until March 2017. The projects aimed to provide direct benefits to local people that received project support as well as indirect benefits to these peoples’ households and members of the wider community.

The ECRP targeted households that are considered particularly vulnerable in the Malawi context: female-headed, elderly, extremely resource-poor, and those with disabled or chronically ill adults (Ibid.). It professed to take measures to ensure that these households could participate in project activities alongside other local people. This represents a contractarian approach to distributive justice.

Extremely resource-poor households were considered by the projects to be particularly vulnerable because they lack the material assets to adapt to climate and development stresses and shocks (Ibid.). Elderly, disabled and chronically ill people were considered to lack the physical capabilities to do so (Ibid.). Women fare worse than men against a range of socio-economic indicators in Malawi (GoM 2006), meaning female-headed households are also considered a particularly vulnerable group.

**Fig. 2:**
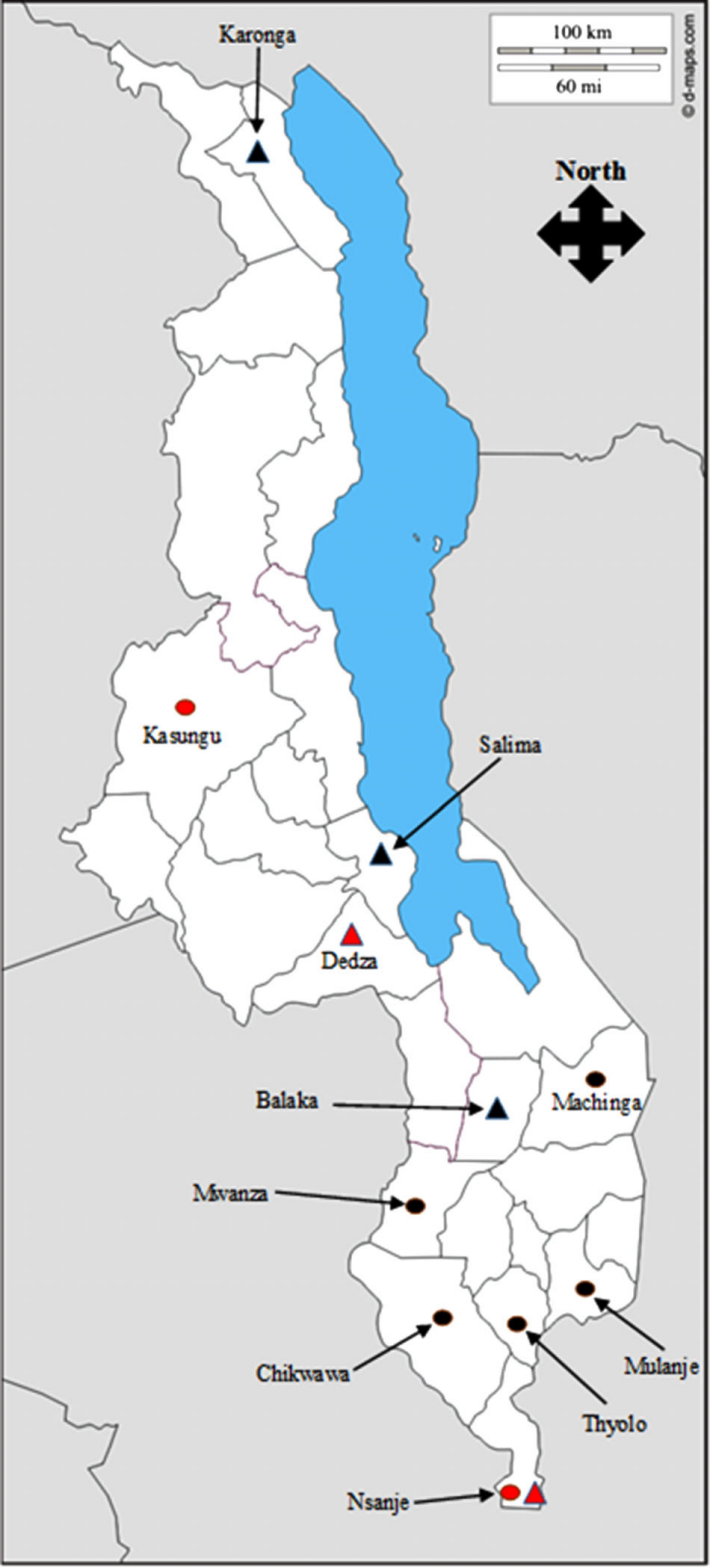
Districts targeted by ECRProject (*circles*) and DISCOVER (*triangles*) projects. Study districts are highlighted in red. Adapted from D-maps (2016)

Research was conducted in three ECRP target districts: Kasungu (ECRProject), Dedza (DISCOVER) and Nsanje (both projects) (Fig. 2). Dedza and Kasungu are both in Malawi’s Central Region and have similar socio-economic characteristics and comparable average rainfall patterns (MVAC 2005). Nsanje is located at Malawi’s southern-most point and is considered to have a lower socio-economic status than Dedza and Kasungu (Ibid.). It is one of the most climate vulnerable districts in Malawi and afflicted by more regular and severe floods and droughts than other study districts (Ibid.).

Two study villages were chosen in each district. Project field workers helped ensure that villages comprised similar household numbers; were geographically close to each other; and implemented similar project activities. In Dedza and Kasungu, two villages where households had, on average, different average levels of resource wealth (an important indicator of vulnerability in Malawi) were purposively chosen. This facilitated consideration of whether and how households’ experiences of project outcomes differed accordingly.

### Framework Development

**Fig. 3:**
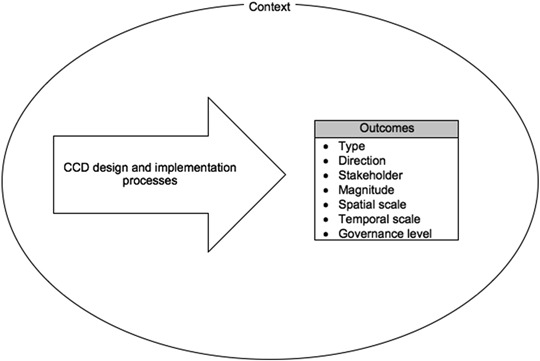
A framework for evaluating CCD project outcomes

A framework was developed to evaluate CCD project outcomes (Fig. 3). A systematic literature review was conducted on English language, peer-reviewed literature to identify parameters for classifying project outcomes. Methods of Ford et al. (2011) were adopted to guide the systematic review process and identify seven parameters: (i) type; (ii) direction; (iii) stakeholder; (iv) magnitude; (v) governance level; (vi) spatial scale; and (vii) temporal scale. Table 1 summarises the results of the systematic literature review and defines outcome parameters (see Online Appendix B for supporting references).

**Table 1:** Descriptions of outcome parameter categories identified using a systematic literature review

Parameter	Description	Summary of supporting evidence
Type	The nature of a project outcome, e.g., development, mitigation, adaptation, auxiliary.	Projects aimed at achieving CCD double—or triple-wins often succeed in achieving development, mitigation, and adaptation outcomes. However, supra-local outcomes, which are indirectly—or un-related to development, mitigation and adaptation, and are not experienced by those stakeholders intended by the project—auxiliary benefits—can also result.
Direction	Whether an outcome is positive—a benefit—or negative—a side-effect.	Many development, mitigation, adaptation and auxiliary outcomes have positive consequences for stakeholders. However, projects have also incurred unintended NSEs.
Stakeholder	Individuals and groups that experience a project outcome.	Benefits and NSEs are often distributed unevenly amongst individuals and groups. Outcome distributions have sometimes been least favourable to the most vulnerable local people, especially women and the resource-poor.
Magnitude	The size or importance of a project outcome.	Relative sizes of outcomes differ widely between projects. This is to be expected because projects are motivated primarily by one or two of CCD’s components (development, mitigation, adaptation), but rarely all three. Analogous project activities may also create outcomes of differing magnitudes when implemented in diverse locations.
Spatial scale	The geographical area in which a project outcome is experienced.	The type, direction, magnitude and stakeholders experiencing of project outcomes may be dissimilar across different geographical areas, jurisdictional spaces and over time. Projects implemented in one location may create benefits or incur NSEs in other places or at other scales. Over time, distributions of NSEs and benefits can change. There is a risk that outcomes experienced as a result of projects will end once implementing organisations’ expertise is withdrawn at the end of project lifespans.
Governance level	The jurisdictional space in which a project outcome is experienced.	
Temporal scale	The timescale over which a project outcome occurs.	

Articles were sought that presented empirical findings related to outcomes of projects aiming to achieve CCD double—or triple-wins in developing countries. The scarcity of literature focussing on triple-wins meant the analysis of articles focussing on both double—and triple-wins was important for capturing a sufficiently broad list of outcome parameters

Articles were located online using the Web of Knowledge electronic database. The following search terms were used:

(“climat* change” or “climat* change adaptation” or “carbon” or “climat* change mitigation”) AND (“development” or “livelihoods”) AND (“project*” or “action*” or “activit*” or “intervention*”) AND (“Africa” or “Asia” or “South America” or “Central America” or “developing nation” or “developing country”)

The search yielded 2122 results. Articles were manually reviewed to filter-out those that did not present empirical findings related to CCD project outcomes, leaving 34 articles for final review.

A realist review approach was adopted, which has an explanatory focus and enabled understanding of why project outcomes differ across parameter categories (Pawson et al. 2005). Review findings highlight that interactions between project design and implementation processes and contextual factors can explain differences. Accordingly, the framework in Fig. 3 considers how project processes shape particular outcomes in the context of socio-ecological and political-economic factors.

### Material Collection and Analysis

Data collection in Malawi took place between September 2014 and May 2015. Information was sought from ECRP stakeholders—individuals, collectives or organisations with the potential to have experienced one or more project outcome. A comprehensive stakeholder analysis was undertaken for each case study project. An initial sample of 10 stakeholders (three donor agency employees; seven NGO employees managing the ECRProject and DISCOVER) was identified through ECRP project design documentation. Additional stakeholders were identified using a snowball sampling approach. Identified stakeholders included: village households; donor agencies; NGOs implementing the projects; the national government of Malawi; and local governments.

Questionnaire surveys (*n* = 457) and semi-structured interviews (*n* = 140) enabled data to be collected from households across study villages. Intra-household distributive justice implications of the projects were beyond the scope of this study. Responses were obtained from all available and consenting households in each village, including those that were not participating in projects. In all cases, the household head or another adult household member was surveyed.

Survey data were analysed to identify project outcomes and categorise them according to outcome parameters set out in Fig. 3. Contextual factors that shaped the outcome characteristics were coded (Babbie 2008). A purposive approach was then adopted to select a sample of surveyed households to be revisited in order to conduct semi-structured interviews.

Semi-structured interviews were also used to gather qualitative data from 32 professional stakeholders: two donor agency employees; 21 NGO employees; one national and eight local government employees. Household and professional stakeholder interviewees were asked about benefits and NSEs they had experienced as a result of projects. Project outcome categories presented in Table 2 were used to structure survey and interview questions to guide data collection. Information on project design and implementation processes and contextual factors that interact to create benefits and NSEs was also sought.

**Table 2:** Categories for classifying project outcome type and direction

Term	Definition
Development benefit	Enhances local people’s capabilities to live the life that they choose (Sen 2001)
Development NSE	Reduces local people’s capabilities to live the life that they choose (Sen 2001)
Mitigation benefit	Could reduce the magnitude of climate change (IPCC 2014)
Mitigation NSE	Could increase the magnitude of climate change (IPCC 2014)
Adaptation benefit	Helps moderate harm of, or exploit beneficial opportunities from, actual or expected climate change impacts (IPCC 2014)
Adaptation NSE	Increases harm of, or prevents exploitation of beneficial opportunities caused by, climate change impacts (IPCC 2014)
Auxiliary project benefit	Any advantageous project outcome that does not fall within ‘development’, “mitigation” or “adaptation” framework categories
Auxiliary project NSE	Any inconveniencing project outcome that does not fall within “development”, “mitigation” or “adaptation” framework categories

Local people’s participation within wealth ranking exercises can help enhance their precision and contextual appropriateness (Chambers 1994). Indicators were developed using a participatory approach (Jefferies et al. 2005) in order to distinguish between responses of “lower-than-average wealth”, “average wealth” and “higher-than-average wealth” households in the context of particular villages. This enabled analysis of the extent to which projects had targeted benefits towards households considered extremely vulnerable owing to the extent of their resource poverty.

Documentary material was collected and analysed. The ECRP mid-term evaluation report produced by independent consultants (LTSI 2014) provided further information on project outcomes in target districts. Both the mid-term evaluation report and the following documents were used to estimate mitigation outcomes that result from projects’ forestry, improved cookstoves and solar light components: CU (No Date); CA (No Date); SA (2015); CDI (2011).

Univariate analysis techniques were used to analyse statistics derived through amalgamating household survey responses within and across villages. CA was used to analyse survey, interview and documentary data (see Babbie 2008). Categories presented in Table 2 were used to classify outcome “type’ and “direction”. Data analysis uncovered four governance levels at which project outcomes were experienced: international; national; district; and household.

Households and professional stakeholders who reported experiencing project outcomes were asked to assess the magnitude of development, adaptation and auxiliary outcomes in interview and survey responses. Stakeholders reporting experience of benefits and/or NSEs were asked to rate outcomes in terms of their perceived importance (positive or negative). A rating scale of 1–3 was used (1 = outcomes had a near-negligible significance for stakeholders; 3 = outcomes had a very significant impact). Mean importance ratings were calculated for each outcome. The mean was calculated as a measure of central tendency because the data were neither skewed nor based on categorical variables. Constant comparison techniques were used to determine how reported project outcomes differed within and between: (a) stakeholder groups, and; (b) different household types (demarcated by wealth categories and whether households were female-headed and/or elderly-headed).

Ratings from stakeholder testimonies are inappropriate for measuring mitigation benefits. Climate inertia and variability make mitigation benefits and NSEs very hard to detect (Tebaldi and Friedlingstein 2013). When successful mitigation occurs, benefits are usually evidenced only several decades after the activities creating these benefits are instigated. Some mitigation activities, especially those involving land-use changes, can also take a long time to yield benefits. Because case study projects only began in 2011, this study took place before most mitigation outcomes had occurred or affected the climate. The magnitude of mitigation outcomes was therefore estimated in terms of tonnes of CO_2_ (*t*/CO_2_) expected to be saved through project activities.

Direct mitigation benefits of solar lighting and improved cookstove activities were estimated by multiplying projected household adoption figures with average carbon savings resulting from product use. No data exist concerning the quality and quantity of biomass cover resulting from ECRP forestry activities; only numbers of households participating in activities have been recorded. Making estimations of possible carbon savings is therefore extremely difficult. The Clinton Development Initiative *Trees of Hope* project, operating in Neno and Dowa Districts in Malawi, monitors carbon savings that result from forestry activities—woodlot regeneration, boundary planting—that were analogous with the ECRP in terms of species planted. Carbon savings are estimated using the Plan Vivo methodology, which is used to accredit projects across Africa, Latin America and the Asia-Pacific region (Plan Vivo 2017). The average expected carbon sequestration per participating smallholder farming household across the 50-year *Trees of Hope* crediting period was calculated (total expected carbon sequestration divided by total households). This number was then multiplied by figures projecting future ECRP household forestry activity participation rates to arrive at estimates of forestry mitigation benefits.

CA was also considered both by ECRP staff (CA No Date) and within the wider literature (Giller et al. 2015) to be able to contribute to carbon savings. Yet, no projects that measure soil carbon sequestration from CA are operational in Malawi. As such, no estimates of enhanced soil carbon storage can be provided, meaning results may underestimate direct mitigation benefits provided by the ECRP. In any case, given the discrepancies in definitions and techniques that are labelled as “CA” (Whitfield et al. 2015), it may be spurious to estimate CA carbon savings based on data from other projects.

## Results

Outcomes experienced by ECRP stakeholders due to their involvement in projects are now presented. The supplementary material accompanying this article includes further detail on, and quotes evidencing, the results. Findings pertaining to different projects, districts and villages mirrored each other, showing little discernible variation. Therefore, presentation of these findings is combined.

### Household Development and Adaptation Benefits

Local people have experienced a range of development benefits from the projects (Table 3; Online Appendix C). However, benefits were only experienced by a minority of households in study villages. Economic development gains, including increased income (135 households of 329 participating in projects) and asset ownership (48/329), were the most often reported development benefits. Projects also contributed to better food security through: enhanced crop yields (149/329); year-round harvesting (44/329); improved food purchasing power (27/329); and better nutrition (18/329). All development benefits were considered by households experiencing them to have had a very significant positive impact on their lives. Hence, although benefits were experienced only by relatively few ECRP participants, for these people they were substantial (Table 3).

**Table 3:** Development and adaptation benefits experienced by households in study villages

Outcome	Main project activities attributed to	Number of reporting households (total participating households in study villages = 329)	Mean importance rating (0 = unimportant, 3 = extremely important)
*Development benefits*			
Increased income	• Village Savings and Loans Associations (VSLAs)	135	3.00
	• Conservation agriculture (CA)		
Improved business opportunities	• VSLAs	19	3.00
	• CA		
Improved asset ownership	• VSLAs	48	2.96
Improved food security (Enhanced crop yields)	• CA	149	3.00
	• Irrigation		
Improved food security (Year-round harvesting)	• Irrigation	44	3.00
	• Seed multiplication schemes		
Improved food security (enhanced food purchasing power)	• VSLAs	27	3.00
More nutritious diet	• Malnutrition training	18	3.00
Improved firewood access	• Forestry and improved cookstoves	18	3.00
Ability to finance better education for children	• VSLAs	7	3.00
Reduced incidence of smoke-related illness	• Improved cookstoves	5	3.00
*Adaptation benefits*			
Reduced vulnerability to dry spells due to improved soil moisture and quality	• CA	81	2.97
Houses, assets and farmland protected from heavy rainfall and flooding	• Forestry	32	2.95
Houses, assets and farmland protected from heavy winds	• Forestry	16	3.00
Ability to grow food throughout the year increases households’ abilities to deal with individual climate shocks	• Seed multiplication	8	3.00
Access to emergency finance enables responses to the consequences of climate shocks	• VSLAs	34	3.00

Adaptation benefits were even less widely reported but they were also considered to have had a very significant positive impact by those experiencing them (Table 3; Online Appendix D). 81 out of 156 households undertaking CA reported that the moisture content and quality of soils on their farmland had improved as a result. They reported that this facilitated adaptation benefits because agricultural productivity is compromised less by dry spells. 32 out of 202 households taking part in forestry activities considered trees to have protected their homes, assets and farmland from heavy rainfall and flooding.

There is evidence that household benefits have multiplied across spatial scales: spreading to non-participating households and non-target villages. One higher-than-average wealth household interviewee in Nsanje suggested that project activities implemented by the ECRProject were being copied by nearby households who did not reside in target villages:

Those people (in surrounding villages) are admiring that our lives are improving. They try to copy the activities, although some activities, like irrigation, are difficult to copy. But VSLAs are not so hard and now they have their own.

Two Dedza households reported that people from neighbouring villages have adopted CA after being impressed by increased crop yields in DISCOVER target villages.

### Global Mitigation Benefits

In contrast to the modest adaptation benefits reported by households, the projects could make a significant global-scale mitigation contribution. All activities that create mitigation benefits have also led to development gains (Tables 3 and 4), generating significant development-mitigation synergies. Table 4 outlines a range of carbon savings that could be made through household adoption of improved cookstoves, solar lights and forestry activities. Findings from the ECRP mid-term evaluation (LTSI 2014) show that adoption of low-carbon technologies and forestry had been under target. Hence, mitigation benefits that would result from projects meeting adoption targets and continuing to follow mid-term evaluation adoption trends are presented.

**Table 4:** Estimated mitigation benefits resulting from adoption of low-carbon technologies and forestry activities under the ECRP

Low-carbon technologies				
Activity	Average yearly CO_2_ saving (*t*/CO_2_)	Average service life (years)	Households adopting	Total CO_2_ saving (*t*/CO_2_)
Improved cookstoves	1.6	3.92	Project target: 55,210	346,279
			Project following mid-term evaluation trends: 16,010	100,420
Solar lights	0.2	3	Project target: 45,841	27,504
			Project following mid-term evaluation trends*:* 5333	3319
Forestry activities				
Performance indicators	Households adopting	Average CO_2_ savings per participating household over a 50-year period under the Trees of Hope Project (*t*/CO_2_)		Projected total CO_2_ savings over a 50-year period under ECRP (*t*/CO_2_)
Project target	58,187	76.92		4,475,744
Mid-term evaluation trends	33,534			2,579,435

Data sources: SA (2015); LTSI (2014); CA (No Date); CU (No Date); CDI (2011); personal communication with Hestian Innovation

### Issues Hindering Benefit Creation

Various issues hinder the translation of project activities into household benefits (Table 5; Online Appendix E). They may also undermine the longevity of project benefits now that the ECRP has formally ended; compromising households’ abilities to keep practising project activities and thereby also undermining possible mitigation benefits. Intractable financial poverty and poor market access were detrimental to the performance of a range of activities. Revenue shortages across Malawi also meant that extension support for ECRP projects was patchy. Unreliable extension hindered projects’ ability to deliver CCD benefits and, now that project field support has been withdrawn, could be detrimental to benefit longevity.

**Table 5:** Issues that hinder the translation of project activities into CCD benefits and threaten their longevity

Issue	Description	Reported impact(s)	Reported by
*Agricultural activities*			
Negative perceptions of CA	• Traditional agricultural practices involve farmers digging the soil	• CA dis-adoption	9 households, 1 NGO employee
	• Households are teased and abused by other villagers for participating in CA, which requires minimum soil tillage	• Only small areas of land committed to CA	
Delayed CA benefits	• Organic nutrients are fully absorbed into soils only after two to 3 years, leading to delayed development and adaptation benefits		2 households, 2 NGO employees
Poor fertiliser access	• Application of synthetic fertilisers can help offset delayed CA benefits but household access is poor	• Poor harvests	7 households, 1 NGO employee
		• CA dis-adoption	
Pest attacks	• Insects and weeds damage crops and organic soil cover	• Poor harvests	9 households
		• CA benefits lost	7 households, 3 NGO employees
Co-existence with livestock and other animals	• Goats and baboons eat and damage crops and organic soil cover		
Expense of irrigation and seed multiplication upkeep	• Households cannot afford to replace (a) irrigation infrastructure when it breaks down, and (b) seeds required for multiplication schemes	• Irrigation and seed multiplication benefits lost	6 households, 1 donor employee; 1 NGO employee
Poor market access	• Households—especially residents of remote villages—have inadequate access to suitable markets for selling cash crops	• Agricultural activity benefits reduced or lost	2 households, 1 NGO employee
Extreme weather events	• Droughts and severe dry spells compromise benefits of agricultural activities	• Agricultural activity benefits reduced or lost	26 households, 2 NGO employees
	• Heavy rains destroy crops and organic soil cover and undermine CA soil fertility gains	• Seed multiplication compromised	
	• Heavy rain can lead to waterlogging when CA is practised	• Poor harvests	
		• CA dis-adoption	
*Livestock production*			
Prioritisation of short-term benefits	• Livestock participants give the offspring of the animals that they receive to other households in order for associated benefits to spread throughout villages. However, households sometimes eat or sell livestock shortly after passing on offspring in order to access food and income quickly or in response to climate and development shocks	• Sustainable livestock production benefits (e.g., access to manure, goats milk) lost	14 households
*Forestry*			
Communal, non-immediate benefits	• Participating and non-participating households benefit similarly from afforestation. Households are disillusioned about participating in afforestation, which does not yield immediate benefits, for “free”. They would like to receive additional, immediate benefits in return for their labour	• Limited participation in forestry activities	3 households, 1 NGO employee
		• Forestry benefits reduced or foregone	
Extreme weather events	• Dry spells and drought mean tree seedlings do not receive enough waterHeavy rains and floods damage and destroy trees	• Forestry benefits reduced or foregone	8 households
*VSLA*			
Drop-outs	• VSLA members struggle to pay back loans and are forced to withdraw from groups	• Reduced availability of loans	32 households
Challenges for doing business	• Financial poverty translates into limited markets for new businesses	• Business profits limited	4 households
	• Low education levels limit innovation that is required for business success		
*Low-carbon technologies*			
Limitations of market-based approaches	• Financial poverty in ECRP target villages makes it difficult for households to afford products	• Low affordability and lack of awareness reduces markets for solar products and cookstoves	4 NGO employees
	• Unsensitised households in non-ECRP target villages are unaware of products	• Few have capital required to become solar entrepreneurs	
Opportunity costs of improved cookstove production	• Other livelihood options are more profitable than cookstove production	• Stove production eschewed in favour of other livelihood activities	3 NGO employees
	• DISCOVER pledged to top-up income from cookstove sales with money obtained from carbon credit sales, but this has yet to materialise		
Cheaper solar products available	• Cheaper solar products than those sold under ECRP are available	• Products unaffordable	4 NGO employees
	• Poor quality of alternative products deters investments in solar	• Solar entrepreneurship too capital intensive	
*ALL ACTIVITIES*			
Patchy extension worker services	• Extension service capacity across Malawi is patchy. Reduced training and policing of project activities could create problems in villages without sufficient support once ECRP comes to an end	• Households receive insufficient technical advice	12 households, 2 NGO employees
		• Reduced incentives to spread project resources within villages	

Project activities often failed to help people to adapt to current or future climate change because their practice was hindered by extreme weather events. Ecosystem-based activities (e.g., agricultural activities, forestry) were particularly sensitive to climate shocks. Projects are predominantly framed in terms of their pursuit of development and adaptation goals, with activities prioritised that also create mitigation benefits. There is a mismatch between this framing and the outcomes reported by project stakeholders. Participating households received nearly four times as many development benefits (1.8) as adaptation benefits (0.48), on average. That extreme weather events acutely hinder the translation of project activities into adaptation benefits provides one explanation for this. Activities’ climate sensitivity could also undermine current and future development benefits, as well as mitigation gains that they stand to create over time.

In some cases, implementation issues have been reduced or overcome. For example, some households hold negative perceptions of CA because they contrast with traditional farming practices. However, a village extension multiplier in one Nsanje village suggested that negative perceptions of CA have softened when those holding them witness superior crop yields achieved by CA adopters. Non-adopters have reportedly “been impressed” and have “said they will re-join”. Likewise, project field workers have reduced instances of crops being destroyed by livestock. One average wealth Kasungu household explained that “we have been taught a new method [by field officers]…which involves tying together stalks and looking after them at home…this means goats cannot get to them”. NGOs have also encouraged villages to develop bylaws to prevent livestock from damaging crops. Other implementation issues (e.g., activities’ climate sensitivity, those linked to financial poverty and poor market access) appear more persistent.

### Negative side-Effects

Issues hindering the performance of project activities have led to NSEs (all receiving mean importance rating scores of between 2.88 and 3.00) for local people (Online Appendix F). Nine households reported that they had lost money as a result of participating in VSLAs. They reported that financial poverty often translated into limited output markets and meant small businesses established through VSLA loans were unprofitable. For example, one elderly-headed, female, lower-than-average household head in Kasungu complained that “debt collectors took two goats from me while my son paid his debt but I never got them back”. Three households in one Nsanje village perceived that increased resource wealth resulting from ECRP activities had led to greater instances of theft. Practising project activities in the context of extreme weather events has also led to NSEs. For instance, three households considered that, under conditions of heavy rainfall, CA led to waterlogged fields and reduced crop yields.

Increased inequality within target villages was the most frequently reported NSE (by 16 households). Analysis of how project benefits were distributed amongst different household types supports these testimonies. On average, 2.27 benefits were experienced by households participating in ECRP project activities. However, higher-than-average wealth households experienced significantly more development and adaptation benefits than any other household type: 3.08 total benefits on average, compared with 2.20, 2.30, 1.97 reported by average wealth, elderly-headed and lower-than-average wealth households, respectively. Female-headed households received the fewest benefits overall (1.91), and the fewest adaptation benefits (0.31). Female-headed and lower-than-average wealth households experienced the fewest development benefits (1.6), on average.

One NGO employee considered that the processes by which livestock participants were chosen created NSEs for the extremely resource-poor. In some DISCOVER villages, households were asked to spend time and resources building corrals to show they were “capable” of keeping livestock. Some took loans to afford construction materials. However, livestock were rarely distributed to extremely resource-poor households. Livestock activities operated on a “pass-on” principle whereby initial participants gave the offspring of the animals that they receive to other households in order for associated benefits to spread throughout villages. NGOs worried that resource-poor households might: (a) sell livestock for immediate cash benefits; and/or (b) lack the capabilities to look after animals properly. Hence, they were concerned that distributing animals to these people might compromise the pass-on principle. According to the NGO employee: “there have been cases whereby we say no, you have the corral but you are not fit”.

Growing inequality has occurred despite projects targeting the most vulnerable households (CU No Date; CA No Date). Local people were not involved in the decision to target benefits towards the most vulnerable but they agreed with the principle of doing so. There was broad consensus amongst household interviewees that all residents within study villages deserve assistance to reduce their vulnerabilities. This was attributed to residents’ widespread inability to fulfil their basic needs: “everyone should receive the benefits (from projects). These are the basic needs for everyone and all should be considered. Weather problems affect us all” (Nsanje higher-than-average wealth household interviewee). Respondents believed that certain groups require particular attention, including: the resource-poor, elderly-headed households, the disabled, the chronically-ill, women and orphans. For example, one household (average wealth, elderly headed) in an Nsanje village commented:

Very poor and disabled people should benefit first because they need most help…The very poor should also receive help to deal with difficult weather conditions first because they have few sources of livelihood. It would also help reduce the gap between the rich and the poor.

Respondents were not questioned on whether they would still support a contractarian distributive justice approach if this also meant that they would receive fewer benefits from the projects. However, findings suggest that increased inequality being experienced in study villages as a result of the ECRP is both at odds with conceptions of distributive justice held by local people and espoused by the projects themselves.

### Auxiliary Benefits

In addition to producing CCD outcomes, projects have also generated auxiliary benefits for stakeholders operating above the village level. For example, access to financial resources was reported as an auxiliary benefit by NGO employees. Local government, NGO and donor agency employees considered that various benefits (improved capacities, innovativeness, reputations, access to resources, lobbying influence, organisational cohesion) have been experienced by their employer organisations. Professional stakeholders unanimously agreed with one NGO employee who considered that many of these benefits would “last beyond the lifespan of the project and inform future work”. All auxiliary benefits received average importance rating scores between 2.50 and 3.00.

Overall, the ECRP has produced patterns of benefits and NSEs that differ across geographical scales and governance levels. Outcomes are distributed unevenly between stakeholder groups and will change over time. CCD triple-wins are being achieved, but they do not represent the full range of outcomes produced.

## Discussion

To date, an absence of suitable evaluation has been a constraint to improved understandings of CCD project outcomes. Our case study analysis of ECRP projects across Malawi shows that holistic understanding of CCD outcomes enables exploration of its distributive justice implications relative to principles of outcome fairness upon which interventions are premised.

We analysed qualitative stakeholder testimonies, which was appropriate because rich local knowledge provides detail that purely statistical evaluations cannot elucidate (Marin 2010). Considering detailed testimonies was important for elucidating contextual factors that shaped project outcomes. Understanding outcomes that are perceived and experienced by target populations also matters because local acceptance is critical for the successful rollout of CCD (Anton et al. 2014).

However, evaluating outcomes based upon stakeholder testimonies has limitations. For example, local people may have been wary of revealing NSEs for fear of damaging their relationships with implementing NGOs. Local people were briefed by project staff about the benefits they should expect to receive. This may have led them to over-attribute benefits to project activities and overlook possible alternative explanations. Other outcomes may not have been perceived by stakeholders to have resulted from projects and may be under-reported. Further research that combines and triangulates qualitative and quantitative data could overcome some of these limitations and would make a valuable addition to the CCD literature.

Overall, our results point to two key findings that resonate with the CCD literature and are now discussed in turn:

1. Outcome patterns created by projects do not reflect the popularised depiction of CCD;
2. Community-based CCD may be insufficient to enable contractarian distributive justice.

### Outcome Patterns Created by Projects do not Reflect the Popularised Depiction of CCD

ECRP projects were presented by implementing partners as achieving development and adaptation benefits through activities that contribute to carbon savings or are carbon neutral (Mitchell and Maxwell 2010). Yet our analysis suggests that projects created a range of NSEs (e.g., increased inequality within villages, decreased resource wealth, increased crime) and auxiliary benefits (e.g., improved capacities, innovativeness, reputations, access to resources, lobbying influence and organisational cohesion experienced by professional stakeholder organisations) alongside triple-wins.

**Fig. 4:**
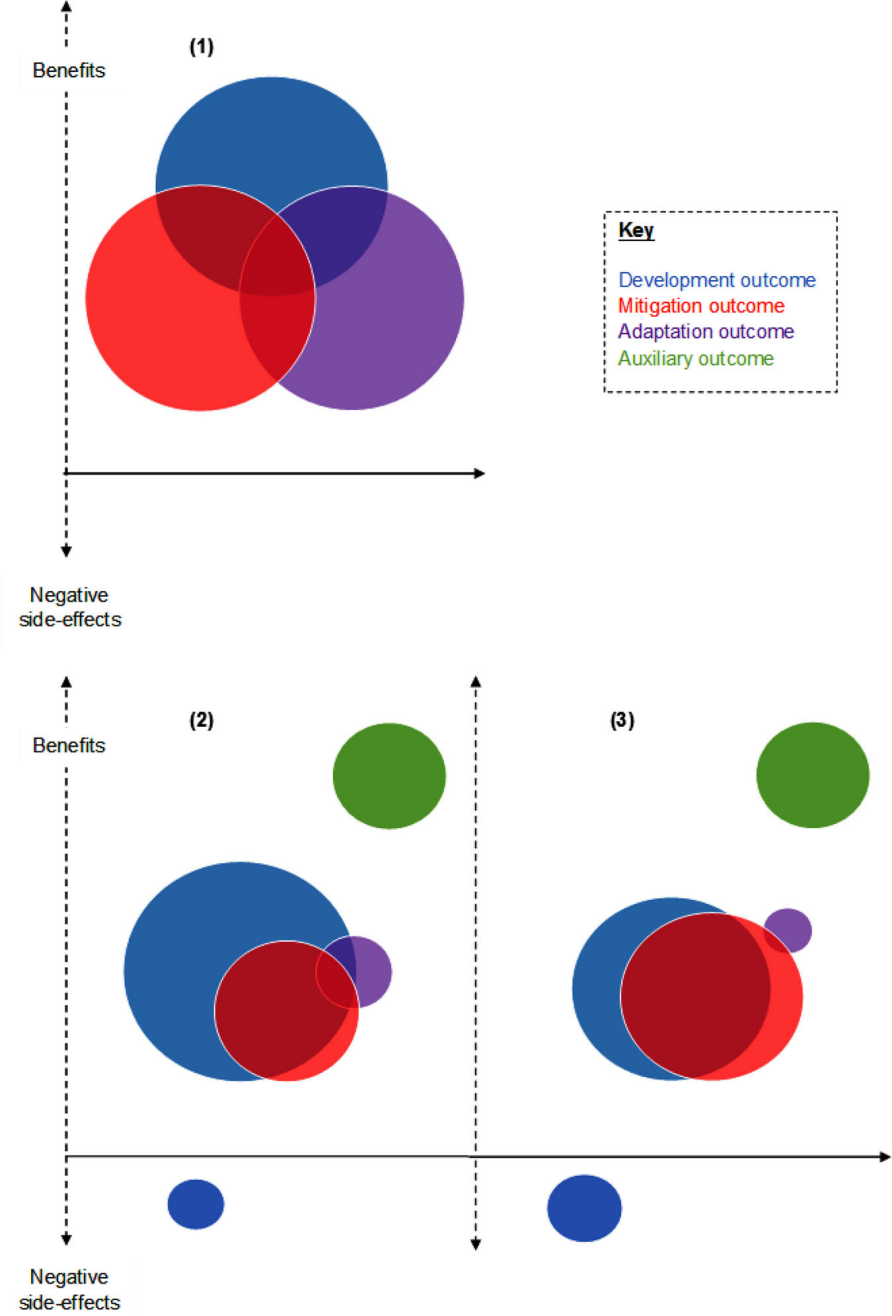
A comparison of the popularised depiction of CCD outcomes (1) with patterns of benefits and NSEs created by the ECRP during (2) and beyond (3) project lifespans. Descriptions of, and differences between, (1), (2), and (3) are outlined in the text

ECRP project outcomes will change over time because most mitigation benefits have yet to develop and a range of issues threaten the sustainability of project activities. Outcomes were also experienced differently by diverse individuals and groups operating across dissimilar geographical locations and governance levels. For instance, households experienced development and adaptation outcomes but mitigation benefits will be experienced at the global-scale and auxiliary benefits were experienced by professional stakeholders at supra-local levels. Within villages, benefits and NSEs were distributed unevenly between household types. Our findings build on the work of Tompkins et al. (2013) who criticise the popularised depiction of CCD because it fails to draw attention to the full range of outcomes that might be created. Fig. 4 illustrates this point, comparing the outcomes created by ECRP projects during and beyond the projects’ lifespan with the popularised depiction of CCD.

Although projects created CCD triple-wins, the balance between development, mitigation and adaptation benefits produced by the ECRP was at odds with its framing. Although presented predominantly in terms of development and adaptation objectives, our findings suggest that projects stand to further local-level development progress in Malawi and make a significant global-scale mitigation contribution. However, expected project adaptation benefits in ECRP villages may have been over-estimated. Low carbon technologies and forestry activities implemented by projects could avoid and/or sequester up to 2683,174*t*/CO_2_ over a 50 year period if household participation continued along mid-term evaluation trajectories. Further carbon savings might result from ECRP CA activities but estimating these carbon savings was beyond the scope of this study. Because activities that create mitigation benefits also led to development gains (e.g., forestry activities improved firewood access, improved cookstove use reduced smoke-related illness), ECRP activities generated significant development-mitigation synergies (Fig. 4).

Mitigation estimates do not account for issues that could reduce the sustainability of benefits over time (e.g., climatic limits, patchy extension services). These issues are likely to compromise estimates of forestry mitigation benefits because they are projected over a 50-year period. By contrast, carbon savings from improved cookstove and solar light adoption are only measured over short product lifespans (3–4 years).

ECRP projects made only a modest contribution to adaptation in study villages (Fig. 4). Curtailed scientific understandings of climate change amongst local people related to limited education levels and traditional belief systems (Simelton et al. 2013) may have meant adaptation benefits were underreported. Yet, modest adaptation benefits are more likely explained by the fact that activities implemented by ECRP projects are themselves very sensitive to climate shocks and stresses. In some cases, practising project activities in the context of extreme weather events led to NSEs for local people.

If development progress generated by the projects is maintained, the ECRP might help build local people’s adaptive capacity with which to respond to future climate impacts (Lemos et al. 2007). This would create areas of overlap between development and adaption that are promoted by CCD (Mitchell and Maxwell 2010). However, projects struggled to meaningfully alleviate climate change impacts that already threaten development progress. This, in turn, undermines the likelihood of local adaptive capacity being built.

Ecosystem-based activities are increasingly used to pursue CCD goals (Munang et al. 2013). Yet, they were particularly sensitive to climate shocks under the ECRP. This finding is mirrored by wider research across the developing world (Schwilch et al. 2014) and suggests there may be climatic limits to ecosystem-based CCD. Benefits may be time-bound and activities may even increase local people’s vulnerabilities—maladaptation (Barnett and O’Neill 2010). In particular, the adaptation benefits that can be created by ecosystem-based CCD may have been over-estimated. Given the speed at which they are being adopted by practitioners, possible climatic limits to ecosystem-based activities’ CCD benefits represents a pressing research gap.

Over-estimation of adaptation benefits under CCD may even extend beyond ecosystem-based approaches. This is because projects professing to pursue adaptation goals are frequently composed of re-packaged rural development activities that are fundamentally unaltered (Ireland 2012). Repackaging is incentivised by NGOs’ desire to attract development funding that is increasingly being channelled into adaptation finance (Ibid.). Under the ECRP, non-financial auxiliary benefits (e.g., enhanced reputations, innovation and lobbying influence) that accrued to NGOs may also have incentivised repackaging.

### Community-Based CCD may be Insufficient to Enable Contractarian Distributive Justice

The plurality of values and interests that coexist and conflict in the context of CCD mean it is impossible to underpin the concept with a universal standard of distributive justice (Fisher 2015). Ideally, the particular nature of distributive justice adopted through specific interventions should negotiate between these interests (Paavola and Adger 2006). Given that CCD is being implemented to reduce the vulnerabilities of local people, the principles of procedural justice require that their voices must be heard (Schlosberg 2007). The contractarian standard of distributive justice underpinning the ECRP was determined without local involvement. However, our results show that local people were supportive of project intentions to provide development and adaptation benefits to all households within target villages whilst focussing project activities and associated benefits on particularly vulnerable households.

In contrast to its stated approach, ECRP outcomes were perceived to perpetuate local inequalities and provided least benefit to underprivileged household types. Lower-than-average wealth and female-headed households, who are considered amongst the most vulnerable households in Malawi (GoM 2006), received the fewest benefits of all household types. Similar findings have also resulted from evaluations of other projects that pursue CCD in developing countries (Mathur et al. 2014).

A paradox of vulnerability appeared to compromise the fulfilment of a contractarian standard of distributive justice through ECRP projects. The same socio-economic conditions that led local people to be labelled as “the most vulnerable” also prevented them from reducing their vulnerabilities. The projects’ community-based approach was premised on the idea that local people have the skills, knowledge and resources to further their own development. However, Wood et al. (Under Review) show that limited access to human and material resources (e.g., finance, time, health) obstructed the participation of lower-then-average wealth, female-headed and elderly-headed households in the ECRP. Consequently, these groups accrued fewer project benefits than other household types. This was exacerbated in cases where traditional leaders used their authority to manipulate project implementation processes in order to monopolise participatory opportunities and benefits for themselves and their friends and families (Ibid.). Traditional leaders, which include village heads, are highly respected in Malawi and have significant decision-making authority within rural areas (Bryceson and Fonseca 2006). Findings point to a reciprocal relationship between procedural and distributive justice: something that is theorised (e.g., Schlosberg 2007) but infrequently supported by empirical evidence.

Even when households were able to meaningfully participate in projects, benefits accrued were curtailed. Issues associated with wider patterns of underdevelopment in Malawi that are beyond the control of local people obstructed development and adaptation progress. Poor market access and availability meant households were often unable to sell crops produced through agricultural activities and make profitable investments using VSLA loans (see also Bele et al. 2014). Consequently, they have been unable to escape from intractable financial poverty, which itself compromised the performance of project activities. These conditions even resulted in the creation of development NSEs (e.g., when financial losses resulted from poor VSLA loan payback). Patchy extension support also hinders local people’s abilities to achieve CCD benefits in the present, both in Malawi and elsewhere (Wright et al. 2014). Implications will be even more detrimental now that project field support has been withdrawn, threatening benefit longevity (Orchard and Stringer 2017).

Community-based approaches are becoming institutionalised alongside contractarian distributive justice principles within climate and development practice (Reid 2015). However, evidence concerning whether and to what extent these activities can contribute to CCD benefits at all, let alone direct those benefits towards the most vulnerable, is highly contested. Existing evidence is often politicised, circumstantial and outpaced by success claims (Whitfield et al. 2015; Brau and Woller 2004; Urmee and Gyamfi 2014). Our findings suggest that community-based CCD projects may be insufficient for easing the plight of the most vulnerable people. These findings chime with criticisms of community-based projects that aim to achieve single—or double-wins across development, mitigation and adaptation (Dodman and Mitlin 2013).

Contending with resource scarcity and structural issues that condition local vulnerability will require community-based CCD projects to create links with development efforts across levels and scales. Linking very vulnerable households identified through projects with social protection schemes, such as food and cash transfers, could help enable their involvement. There is an acute need for projects to identify enabling factors that help overcome non-material barriers to participation. Collaborative working between donors, NGOs and governments (national and local) will be crucial for contending with particularly onerous structural issues that condition vulnerabilities.

## Conclusion

This article has addressed the underdeveloped evidence base around outcomes created by CCD projects and their links to distributive justice. A framework was developed that enables CCD outcome evaluation across seven parameters. The framework was used to analyse new empirical data in order to evaluate outcomes that result from the implementation of two donor-funded projects in Malawi that pursue CCD.

Our research shows that ECRP projects produced multi-level patterns of benefits and NSEs that differ across time and space and which were sometimes misaligned with both popular depictions of CCD and the projects themselves. Outcomes have had diverse consequences for different individuals and groups and are at odds with contractarian principles of distributive justice that were espoused by the projects. Our analysis of the ECRP points to a range of outcomes that were not identified through the mid-term evaluation (LTSI 2014). For example, the mid-term evaluation did not capture the increased inequality within target villages that we found to result from projects (a NSE). This showcases the benefit of evaluating CCD using frameworks that consider the full range of outcomes that it stands to create.

Findings point to a need for greater transparency in terms of: (a) the outcomes that CCD approaches can realistically achieve; (b) who these outcomes stand to benefit; and (c) at whose expense. Only then can the expediency of pursuing CCD be properly evaluated. In particular, this would allow the utility of pursuing CCD triple-wins to be assessed relative to the merits of pursuing single or double-wins.

Projects are increasingly utilising ecosystem—and community-based activities in order to pursue CCD goals. However, our results call into question (a) the suitability of ecosystem-based activities for furthering adaptation progress, and (b) the complementarity between community-based activities and efforts to target CCD benefits at the most vulnerable. A strengthened evidence base is required to ensure that these approaches are able to meet CCD goals and further distributive justice.

## Electronic supplementary material

Supplementary Appendix A

Supplementary Appendix B

Supplementary Appendix C

Supplementary Appendix D

Supplementary Appendix E

Supplementary Appendix F

Supplementary Appendix G

## References

[CR1] Adger WN, Brown K, Nelson DR, Berkes F, Eakin H, Folke C, Galvin K, Gunderson L, Goulden M, O’Brien K (2011). Resilience implications of policy responses to climate change. Wiley Interdisciplinary Rev Clim Change.

[CR2] AidData. (2016) *Donor Dependence, Donor Withdrawal: Implications of Malawi’s Cashgate Scandal* [Online]. [Accessed 29 November 2016]. http://aiddata.org/blog/donor-dependence-donor-withdrawal-implications-of-malawis-cashgate-scandal

[CR3] Anton B, Cambray A, Dupar M, Westerlind-Wigstroem A, Gogoi E. (2014) *Close to home: subnational strategies for climate compatible development*. CDKN Working Paper. CDKN.

[CR4] Ayers J, Forsyth T (2009). Community-based adaptation to climate change. Environ Sci Policy Sustain Dev.

[CR5] Babbie E (2008). The basics of social research.

[CR6] Barnett J, O’Neill S (2010). Maladaptation. Global Environ Change.

[CR7] Barrett S (2013). Local level climate justice?. Global Environ Change.

[CR8] Bele MY, Sonwa DJ, Tiani AM (2014). Local communities vulnerability to climate change and adaptation strategies in Bukavu in DR Congo. J Environ Dev.

[CR9] Brau JC, Woller GM (2004). Microfinance: a comprehensive review of the existing literature. J Entrep Financ.

[CR10] Broto VC, Ensor J, Boyd E, Allen C, Seventine C, Macucule DA (2015). Participatory planning for climate compatible development in maputo.

[CR11] Bryan E, Ringler C, Okoba B, Koo J, Herrero M, Silvestri S (2013). Can agriculture support climate change adaptation, greenhouse gas mitigation and rural livelihoods?. Clim Change.

[CR12] Bryceson DF, Fonseca J (2006). Risking death for survival: peasant responses to hunger and HIV/AIDS in Malawi. World Dev.

[CR13] Chambers R (1994). The origins and practice of participatory rural appraisal. World Dev.

[CR14] Christian AID (CA). No Date. Enhancing Community Resilience Project Design Document.

[CR15] Clinton Development Initiative (CDI). (2011) Project Design Document for the Trees of Hope Plan Vivo Project.

[CR16] Concern Universal (CU). ND. *DISCOVER Project Design Document*.

[CR17] D-MAPS. (2016) *Republic of Malawi [online]*. [Accessed 24 May 2016]. http://www.d-maps.com/carte.php?num_car=4778&lang=en.

[CR18] Department for International Development (DFID). No Date. Enhancing Community Resilience Programme Summary Business Case.

[CR19] Dodman D, Mitlin D (2013). Challenges for community‐based adaptation: discovering the potential for transformation. J Int Dev.

[CR20] Dyer JC, Leventon J, Stringer LC, Dougill AJ, Syampungani S, Nshimbi M, Chama F, Kafwifwi A (2013). Partnership models for climate compatible development. Resources.

[CR21] Ellis K, Cambray A, Lemma A. (2013) *Drivers and Challenges For Climate Compatible Development*. CDKN Working Paper.

[CR22] Fisher S (2015). The emerging geographies of climate justice. Geogr J.

[CR23] Ford JD, Berrang-Ford L, Paterson J (2011). A systematic review of observed climate change adaptation in developed nations. Clim Change.

[CR24] Gaillard J-C (2010). Vulnerability, capacity and resilience. J Int Dev.

[CR25] Giller KE, Andersson JA, Corbeels C, Kirkegaard J, Mortensen D, Erenstein O, Vanlauwe B (2015) Beyond conservation agriculture. Plant Science, doi:10.3389/fpls.2015.00870 10.3389/fpls.2015.00870 PMC4623198 26579139

[CR26] Government of Malawi (GoM). (2006) Malawi’s national adaptation programmes of action. Government of Malawi, Lilongwe.

[CR27] Hulme M (2011). Why we disagree about climate change.

[CR28] Huxham M, Emerton L, Kairo J, Munyi F, Abdirizak H, Muriuki T, Nunan F, Briers RA (2015). Applying climate compatible development and economic valuation to coastal management. J Environ Manage.

[CR29] Intergovernmental Panel on Climate Change (IPCC). (2014) Glossary. In: IPCC, ed. Climate change 2014: impacts, adaptation, and vulnerability. Cambridge University Press, Cambridge

[CR30] Ireland P (2012). Climate change adaptation: business-as-usual aid and development or an emerging discourse for change?. Int J Dev Issues.

[CR31] Jefferies D, Warburton H, Oppong-Nkruma K, Freduh Antoh E (2005) *Wealth Ranking Study of Villages in Peri-Urban Areas in Kumasi, Ghana [online]*. [Accessed]. https://www.reading.ac.uk/ssc/n/resources/Docs/QQA/cs6_kuma.pdf

[CR32] Klinsky S, Dowlatabadi H (2009). Conceptualizations of justice in climate policy. Climate Policy.

[CR33] Liu F (2010). Environmental justice analysis.

[CR34] Lemos MC, Boyd E, Tompkins EL, Osbahr H, Liverman D (2007) Developing adaptation and adapting development. Ecol Soc. 12(2):26

[CR35] LTS International (LTSI). (2014) Enhancing community resilience programme: mid-term evaluation. Edinburgh.

[CR36] Malawi Vulnerability Assessment Committee (MVAC). (2005) Malawi baseline livelihood profiles, Lilongwe, Malawi

[CR37] Mansuri G, Rao V (2004). Community-based and-driven development: a critical review. World Bank Res Obs.

[CR38] Marin A (2010). Riders under storms. Global Environ Change.

[CR39] Mathur VN, Afionis S, Paavola J, Dougill AJ, Stringer LC (2014). Experiences of host communities with carbon market projects. Climate Policy.

[CR40] McDermott M, Schreckenberg K (2009). Equity in community forestry: insights from North and South. Int Forestry Rev.

[CR99] McDermott M, Mahanty S, Schreckenberg K (2012) Examining equity: a multidimensional framework for assessing equity in payments for ecosystem services. Environ Sci Policy 1–12, doi:10.1016/j.envsci.2012.10.006.

[CR41] Mitchell T, Maxwell S (2010). Defining climate compatible development.

[CR42] Munang R, Thiaw I, Alverson K, Mumba M, Liu J, Rivington R (2013). Climate change and Ecosystem-based Adaptation. Curr Opinion Environ Sustain.

[CR43] Nunan F (2017) Making climate compatible development happen, Routledge, London

[CR45] Orchard S, Stringer LC (2017) Challenges to polycentric governance of an international development project tackling land degradation in Swaziland. Ambio. 45(7): 796–807 10.1007/s13280-016-0791-8 PMC5055482 27272347

[CR46] Osbahr H, Twyman C, Adger WN, Thomas DSG (2010) Evaluating successful livelihood adaptation to climate variability and change in southern Africa. Ecol Soc. 15(2):27

[CR47] Oxford Poverty and Human Development Institute (OPHI). (2013) *Country Briefing: Malawi* [Online]. [Accessed 9 January 2014]. http://www.ophi.org.uk/wp-content/uploads/Malawi-2013.pdf?3f40f1

[CR48] Paavola J, Adger WN (2006). Fair adaptation to climate change. Ecol Econ.

[CR49] Pawson R, Greenhalgh T, Harvey G, Walshe K (2005). Realist review. J Health Services Res Policy.

[CR50] Picot H, Moss N (2014) The Sustainable Development Goals: Will they deliver climate compatible development for vulnerable countries? *CDKN Working Paper*

[CR51] Plan Vivo. (2017) Plan Vivo [online] [Accessed 20 April 2017]. http://www.planvivo.org

[CR52] Rahn E, Läderach P, Baca M, Cressy C, Schroth G, Malin D, Van Rikxoort H, Shriver J (2014). Climate change adaptation, mitigation and livelihood benefits in coffee production. Mitig Adapt Strategies Glob Change.

[CR53] Reid H (2015). Ecosystem-and community-based adaptation. Climate Dev.

[CR54] Schlosberg D (2007). Defining environmental justice.

[CR55] Schroeder D, Pisupati B (2010) Ethics, justice and the convention on biological diversity. United Nations Environment Program, Nairobi, Kenya

[CR56] Schwilch G, Liniger H, Hurni H (2014). Sustainable land management practices in drylands. Environ Manage.

[CR57] Sen A (2001). Development as freedom.

[CR58] Simelton E, Quinn CH, Batisani N, Dougill AJ, Dyer JC, Fraser ED, Mkwambisi D, Sallu S, Stringer LC (2013). Is rainfall really changing?. Climate Dev.

[CR59] Solar Aid (SA). (2015) *Our Calculations Explained* [online] [Accessed 25 June 2015]. http://www.solar-aid.org/our-calculations-explained/

[CR60] Stringer LC, Dougill AJ, Dyer JC, Vincent K, Fritzsche F, Leventon J, Falcão MP, Manyakaidze P, Syampungani S, Powell P (2014) Advancing climate compatible development. Reg Environ Change, 14(2):1–13

[CR61] Tebaldi C, Friedlingstein P (2013). Delayed detection of climate mitigation benefits due to climate inertia and variability. Proc Natl Acad Sci.

[CR62] Tompkins EL, Mensah A, King L, Long TK, Lawson ET, Hutton CW, Hoang VA, Gordon C, Fish M, Dyer J. (2013) An investigation of the evidence of benefits from climate compatible development. CCCEP Working Paper No. 124

[CR63] United Nations Development Programme (UNDP). (2015) *Human Development Report* [Online]. [Accessed 9 June 2016]. http://hdr.undp.org/en/2015-report

[CR64] Urmee T, Gyamfi S (2014). A review of improved cookstove technologies and programs. Renew Sustain Energy Rev.

[CR65] Vincent K, Dougill AJ, Dixon JL, Stringer LC, Cull T (2017) Identifying climate services needs for national planning. Climate Policy 17(2):1–14

[CR66] Walzer M (1983). Spheres of Justice.

[CR67] West JJ, Smith SJ, Silva RA, Naik V, Zhang Y, Adelman Z, Fry MM, Anenberg S, Horowitz LW, Lamarque J (2013). Co-benefits of global greenhouse gas mitigation for future air quality and human health. Nat Climate Change.

[CR68] Whitfield S, Dougill AJ, Dyer JC, Kalaba FK, Leventon J, Stringer LC (2015). Critical reflection on knowledge and narratives of conservation agriculture. Geoforum.

[CR69] Wood BT, Quinn CH, Stringer LC, Dougill AJ. Under Review. Implementing climate-compatible development in the context of power. Forum Dev Studies

[CR70] Wright H, Vermeulen S, Laganda G, Olupot M, Ampaire E, JAT M (2014). Farmers, food and climate change. Climate Dev.

