# Supplementary material for: Investigating Climate Compatible Development Outcomes and their Implications for Distributive Justice: Evidence from Malawi
Source: Environ Manage. 2017 May 24;60(3):436–53. doi: 10.1007/s00267-017-0890-8 (PMC5544806; doi:10.1007/s00267-017-0890-8)
Supplement: Supplementary file 1 — Supplementary Appendix A [file 267_2017_890_MOESM1_ESM.docx]

**Appendix A: Projects identified in Malawi which simultaneously pursue development, mitigation and adaptation goals**

| **Project name** | **Sectoral focus** | **Duration** | **Location type** | **Stakeholder partners** | **Funding source(s)** | **Local beneficiaries**^[[1]](#footnote-1)^ |
| --- | --- | --- | --- | --- | --- | --- |
| JANEEMO | Agriculture/ Forestry/ Energy | 2011-2014 | Rural (Dowa and Lilongwe) | International Research Organisation (James Hutton Institute); International Consultancy (Climate Futures); Local NGO (Kusamala) | Scottish Government International Development Fund | 700-800 smallholder farmers and their households |
| Trees of Hope | Forestry/ Agriculture/ Energy | 2007-2050 | Rural (Dowa and Neno Districts) | International NGO (Clinton Development Initiative); Local Government (Dowa and Neno District Agricultural Development Offices); Local Government (Dowa and Neno District Forestry Offices); National Government (Department of Environmental Affairs); International NGO (Energy for Sustainable Development in Africa) | Clinton Development Initiative Grant/ carbon finance (Plan Vivo) | 1,148 smallholder farmers and their households |
| Climate-smart Agriculture for Rural Smallholders in Malawi | Agriculture | 2013-2016 | Rural (Dowa and Lilongwe) | International Research Organisation (James Hutton Institute); International Consultancy (Climate Futures); Local NGO (Kusamala) | Scottish Government International Development Fund | 1,500 farmers and their households |
| Drought Mitigation Through Irrigation and CA Extension (DICE) | Agriculture/ Forestry/ Energy | 2012-2015 | Salima, Dowa, Ntcheu | International NGO (Care International); National Government (through various Ministries and Departments); Local NGO (Total Land Care) | USAID grant | 4,000 households |
| Food, Income and Markets (FIM) II) | Agriculture/ Forestry/ Energy | 2012-2015 | Dowa, Lilongwe, Nsanje, | International NGO (Concern Worldwide); Local Government (Area Development Committees and District Executive Committees); Local NGO (NASFAM); Community-Based Organisations (Names tbc) | Irish Aid Grant; Accenture CSR | 15,000 households |
| Mainstreaming Climate-Smart Agriculture in Solar Irrigation Schemes for Sustainable Local Business Development in Malawi | Agriculture/ Energy | 2013-2015 | Nsanje, Thyolo and Mzimba | International NGO (DanChurchAid); International NGO (Churches Action in Relief and Development); Local NGO (Christian Service Committee of the Churches in Malawi); Local NGO (Kusamala) | Nordic Climate Facility Funding; Other (to be identified) | 15,000 households |
| Kulera Biodiversity Project/ Kulera REDD+ | Forestry/ Agriculture/ Energy | 2010-2013; 2014- | Rural (Nyika-Vwaza complex; Mkuwazi Forest Reserve; Nkhotakota Wildlife Reserve) | Local NGO (Total Land Care); International Research Organisation (Washington State University); International NGO (CARE International); International Consultancy (Terra Global Capital); Donor Agency (USAID); National Government (Department of Forestry) | USAID grant; carbon finance (Climate, Carbon and Biodiversity Standard) | 45,000 households |
| Mountain Biodiversity Increases Livelihood Security (MOBI+LISE) | Forestry/ Agriculture/ Energy | 2010- 2013 | Mulanje and Phalombe | International NGO (Concern Universal); Local Environmental Trust (Mount Mulanje Conservation Trust); Local NGO (Wildlife and Environmental Society of Malawi – WESM) | USAID grant | 53,995 households |

| Enhancing Community Resilience Project (Part of the Enhancing Community Resilience Programme) | Forestry/ Agriculture/ Energy | 2011-2016 | Kasungu, Mangochi, Mwanza, Mulanje,  Thyolo, Chikwawa, Nsanje | International NGO (Christian Aid); International NGO (Action Aid); International NGO (CARE International); Research Organisation (ICRISAT); Local NGO (CADECOM); Local NGO (MALEYSA); International NGO (Heifa International); Local NGO (ADRA Malawi); International NGO (Emmanuel International) | UK, Norwegian and Irish Government (Joint Resilience Unit) grants, USAID grant | 61,000 households (298,500 people) |
| --- | --- | --- | --- | --- | --- | --- |
| DISCOVER  (Part of the Enhancing Community Resilience Programme) | Forestry/ Agriculture/ Energy | August 2011-March 2016 | Nsanje, Dedza, Salima, Karonga, Balaka | International NGO (Concern Universal); International NGO (Cooperazione Internazionale); Local Private Company (Clioma); Goal (International NGO); Self Help Africa (Regional NGO); Solar Aid (Regional NGO); Local Private Company (Cumo); Regional Advocacy Institution (CEPA) | UK, Norwegian and Irish Government (Joint Resilience Unit) grants; Climate Finance aspect being developed for the future | 62,900 households (305,000 people) |
| Fuelling a Greener Future for Farmers in Malawi through the use of Jatropha Curcas | Forestry/ Agriculture | July 2008- | Rumphi, Mzimba, Kasungu, Nkotakota, Dowa, Salima, Lilongwe, Ntcheu,  Dedza, Mangochi,  Machinga, Lilongwe | Local Private Company (Bio Energy Resources Limited) | Carbon Finance (Verified Carbon Standard) | 4,275 smallholder farmers |

1. According to interviewees involved in developing climate change and development projects in Malawi, the average number of individuals within a household is five. Project developers thus typically multiply total household beneficiaries by five in order to determine total individual beneficiaries. [↑](#footnote-ref-1)
