# Supplementary material for: Investigating Climate Compatible Development Outcomes and their Implications for Distributive Justice: Evidence from Malawi
Source: Environ Manage. 2017 May 24;60(3):436–53. doi: 10.1007/s00267-017-0890-8 (PMC5544806; doi:10.1007/s00267-017-0890-8)
Supplement: Supplementary file 2 — Supplementary Appendix B [file 267_2017_890_MOESM2_ESM.docx]

**Appendix B: Descriptions of, and supporting evidence for, outcome parameter categories identified using a systematic literature review**

| **Parameter** | **Description** | **Supporting Reference(s)** | **Summary of Supporting Evidence** |
| --- | --- | --- | --- |
| Type | The nature of a project outcome e.g. development, mitigation, adaptation, auxiliary. | Gong *et al.* (2010); Bacon *et al*. (2014); Brown *et al.* (2011); Ayers and Huq (2009); Atela *et al.* (2015); Boyd *et al.* (2007); Dyer *et al.* (2012); Foster and Neufeldt (2014); Jindal *et al*. (2012); Mathur *et al.* (2014); Mortimer and Grant (2008); Rindefjall *et al.* (2011); Stringer *et al.* (2014); Stringer *et al.* (2012); Subak (2000); Weston *et al.* (2015); Li *et al.* (2015). | Projects aimed at achieving CCD double- or triple-wins often succeed in achieving development, mitigation and adaptation outcomes. However, supra-local outcomes, which are indirectly- or un-related to development, mitigation and adaptation — auxiliary benefits — can also result. |
| Direction | Whether an outcome is positive — a benefit — or negative — a side-effect. | Cavanagh and Benjaminsen (2014); Beyene (2015); Bele *et al.* (2014); Baudoin *et al.* (2014); Bacon *et al.* (2014); Atela *et al.* (2015); Boyd *et al.* (2007); Dressler *et al.* (2012); Erlewein and Nusser (2011); Foster and Neufeldt (2014); Hoffman *et al.* (2015); Jindal *et al.* (2008); Jindal *et al.* (2012); Leventon *et al.* (2015); Li *et al.* (2015); Mathur et al. (2014); Nijnik and Halder (2013); Subak (2000). | Many development, mitigation, adaptation and auxiliary outcomes have positive consequences for stakeholders. However, projects have also incurred unintended NSEs. |
| Recipient | Stakeholders that experience a project outcome. | Atela *et al.* (2015); Erlewein and Nusser (2011); Boyd *et al.* (2007); Dressler *et al.* (2012); Foster and Neufeldt (2014); Hoffman *et al.* (2015); Jindal *et al.* (2008); Jindal *et al.* (2012); Khadka *et al.* (2014); Li *et al.* (2015); Mathur *et al.* (2014); Osbahr *et al.* (2010); Poudel (2014); Stringer *et al.* (2014); Subak (2000); Weston *et al.* (2015). | Benefits and NSEs are often distributed unevenly amongst individuals and groups. Outcome distributions have sometimes been least favourable to the most vulnerable local people, especially women and the resource-poor. |
| Magnitude | The size or importance of a project outcome. | Atela *et al.* (2015); Foster and Neufeldt (2014); Jindal *et al.* (2008); Jindal *et al.* (2012); Li *et al.* (2015); Mortimer and Grant (2008); Stringer *et al.* (2014); Subak (2000); Weston *et al.* (2015); Zhang *et al.* (2015). | Relative sizes of outcomes differ widely between projects. This is to be expected because projects are motivated primarily by one or two of CCD’s components (development, mitigation, adaptation), but rarely all three. Analogous project activities may also create outcomes of differing magnitudes when implemented in diverse locations. |
| Spatial Scale | The geographical area in which a project outcome is experienced. | Baudoin *et al.* (2014); Jindal *et al.* (2012); Li *et al.* (2014); Osbahr *et al.* (2010); Weston *et al.* (2015). | The type, direction, magnitude and recipients of project outcomes may be dissimilar across different geographical areas, jurisdictional spaces and over time. Projects implemented in one location may create benefits or incur NSEs in other places or at other scales. Over time, distributions of NSEs and benefits can change. There is a risk that outcomes experienced as a result of projects will end once implementing organisations’ expertise is withdrawn at the end of these lifespans. |
| Governance Level | The jurisdictional space in which a project outcome is experienced. | Foster and Neufeldt (2014); Jindal *et al.* (2012); Li *et al.* (2014); Mathur *et al.* (2014); Weston *et al.* (2015); Rindefjall *et al.* (2011). |  |
| Temporal Scale | The timescale over which a project outcome occurs. | Baudoin *et al.* (2014); Boyd *et al.* (2007); Foster and Neufeldt (2014); Jindal *et al.* (2012); *Li et al.* (2014); Mathur *et al.* (2014); Stringer *et al.* (2012); Weston *et al.* (2015); Xu *et al.* (2007); Swilch *et al.* (2014). |  |

**References**

ATELA, J.O., P.A. MINANG, C.H. QUINN and L.A. DUGUMA. 2015. Implementing REDD+ at the local level: Assessing the key enablers for credible mitigation and sustainable livelihood outcomes. *Journal of Environmental Management,* **157**, pp.238-249.

AYERS, J.M. and S. HUQ. 2009. The value of linking mitigation and adaptation: a case study of Bangladesh. *Environmental Management,* **43**(5), pp.753-764.

BACON, C.M., W.A. SUNDSTROM, M.E.F. GÓMEZ, V.E. MÉNDEZ, R. SANTOS, B. GOLDOFTAS and I. DOUGHERTY. 2014. Explaining the ‘hungry farmer paradox’. *Global Environmental Change,* **25**, pp.133-149.

BAUDOIN, M-A., A.C. SANCHEZ and B. FANDOHAN. 2014. Small scale farmers’ vulnerability to climatic changes in southern Benin. *Mitigation and Adaptation Strategies for Global Change,* **19**(8), pp.1195-1207.

BELE, M.Y., D.J. SONWA and A.M. TIANI. 2014. Local communities vulnerability to climate change and adaptation strategies in Bukavu in DR Congo. *The Journal of Environment and Development,* **23**(3), pp.331-357.

BEYENE, F. 2015. Incentives and Challenges in Community‐Based Rangeland Management*. Land Degradation and Development,* **26**(5), pp.502-509.

BOYD, E., P. MAY, M. CHANG and F. C. VEIGA. 2007. Exploring socioeconomic impacts of forest based mitigation projects*. Environmental Science and Policy,* **10**(5), pp.419-433.

BROWN, D.R., P. DETTMANN, T. RINAUDO, H. TEFERA and A. TOFU. 2011. Poverty alleviation and environmental restoration using the clean development mechanism*. Environmental management,* **48**(2), pp.322-333.

CAVANAGH, C. and T.A. BENJAMINSEN. 2014. Virtual nature, violent accumulation. *Geoforum,* **56**, pp.55-65.

DRESSLER, W., M. MCDERMOTT, W. SMITH AND J. PULHIN. 2012. REDD policy impacts on idigenous property rights on Palawan Island, the Philippines. *Human Ecology,* **40**(5), pp.679-691.

DYER, J.C., L.C. STRINGER and A.J. DOUGILL. 2012. Jatropha curcas: Sowing local seeds of success in Malawi? *Journal of Arid Environments,* **79**, pp.107-110.

ERLEWEIN, A. and M. NÜSSER. 2011. Offsetting greenhouse gas emissions in the Himalaya? *Mountain Research and Development,* **31**(4), pp.293-304.

FOSTER, K. and H. NEUFELDT. 2014. Biocarbon projects in agroforestry. *Current Opinion in Environmental Sustainability,* **6**, pp.148-154.

GONG, Y., G. BULL and K. BAYLIS. 2010. Participation in the world's first clean development mechanism forest project. *Ecological Economics,* **69**(6), pp.1292-1302.

HOFFMANN, H., G. UCKERT, C. REIF, F. GRAEF and S. SIEBER. 2015. Local biofuel production for rural electrification potentially promotes development but threatens food security in Laela, Western Tanzania. *Regional Environmental Change,* **15**(7), pp.1181-1190.

JINDAL, R., J.M. KERR and S. CARTER. 2012. Reducing poverty through carbon forestry? *World Development,* **40**(10), pp.2123-2135.

JINDAL, R., B. SWALLOW and J. KERR. 2008. Forestry‐based carbon sequestration projects in Africa. *Natural Resources Forum*, **32**(2)*,* pp.116-130.

KHADKA, M., S. KARKI, B.S. KARKY, R. KOTRU and K.B. DARJEE. 2014. Gender equality challenges to the REDD+ initiative in Nepal. *Mountain Research and Development,* **34**(3), pp.197-207.

LEVENTON, J., J.C. DYER and J.D. VAN ALSTINE. 2015. *The private sector in climate governance. Journal of Cleaner Production*, **102**, pp.316-323.

LI, C., H. ZHENG, S. LI, X. CHEN, J. LI, W. ZENG, Y. LIANG, S. POLASKY, M. W. FELDMAN and M. RUCKELSHAUS. 2015. Impacts of conservation and human development policy across stakeholders and scales. *Proceedings of the National Academy of Sciences,* **112**(24), pp.7396-7401.

MATHUR, V.N., S. AFIONIS, J. PAAVOLA, A.J. DOUGILL and L.C. STRINGER. 2014. Experiences of host communities with carbon market projects. *Climate Policy,* **14**(1), pp.42-62.

MORTIMER, N.D. and J.F. GRANT. 2008. Evaluating the prospects for sustainable energy development in a sample of Chinese villages. *Journal of Environmental Management,* **87**(2), pp.276-286.

NIJNIK, M. and P. HALDER. 2013. Afforestation and reforestation projects in South and South-East Asia under the Clean Development Mechanism. *Land Use Policy,* **31**, pp.504-515.

OSBAHR, H., C. TWYMAN, W.N. ADGER and D.S. THOMAS. 2010. Evaluating successful livelihood adaptation to climate variability and change in southern Africa. *Ecology and Society,* ***15****(2).*

POUDEL, D.P. 2014. REDD+ comes with money, not with development. *International Journal of Sustainable Development and World Ecology,* **21**(6), pp.552-562.

RINDEFJÄLL, T., E. LUND and J. STRIPPLE. 2011. Wine, fruit, and emission reductions. *International Environmental Agreements,* **11**(1), pp.7-22.

SCHWILCH, G., H. LINIGER and H. HURNI. 2014. Sustainable land management (SLM) practices in drylands. *Environmental management,* **54**(5), pp.983-1004.

STRINGER, L.C., A.J. DOUGILL, D.D. MKWAMBISI, J.C. DYER, F.K. KALABA and M. MNGOLI. 2012. Challenges and opportunities for carbon management in Malawi and Zambia. *Carbon Management,* **3**(2), pp.159-173.

STRINGER, L.C., A.J. DOUGILL, J.C. DYER, K. VINCENT, F. FRITZSCHE, J. LEVENTON, M.P. FALCÃO, P. MANYAKAIDZE, S. SYAMPUNGANI and P. POWELL. 2014. Advancing climate compatible development. *Regional Environmental Change,* pp.1-13.

SUBAK, S. 2000. Forest protection and reforestation in Costa Rica*. Environmental Management,* **26**(3), pp.283-297.

WESTON, P., R. HONG, C. KABORÉ and C.A. KULL. 2015. Farmer-managed natural regeneration enhances rural livelihoods in dryland west Africa. *Environmental management,* **55**(6), pp.1402-1417.

XU, W., Y. YIN and S. ZHOU. 2007. Social and economic impacts of carbon sequestration and land use change on peasant households in rural China. *Journal of Environmental Management,* **85**(3), pp.736-745.

ZHANG, J., L. XU and X. LI. 2015. Review on the externalities of hydropower. *Renewable and Sustainable Energy Reviews,* **50**, pp.176-185.
