# Supplementary material for: Investigating Climate Compatible Development Outcomes and their Implications for Distributive Justice: Evidence from Malawi
Source: Environ Manage. 2017 May 24;60(3):436–53. doi: 10.1007/s00267-017-0890-8 (PMC5544806; doi:10.1007/s00267-017-0890-8)
Supplement: Supplementary file 3 — Supplementary Appendix C [file 267_2017_890_MOESM3_ESM.docx]

**Appendix C: Development benefits resulting from ECRP, as reported by participating households in study villages**

| **Benefit** | **Main activities attributed to**  **(fractions denote households attributing a benefit to a particular activity relative to those who participated in the activity)^[[1]](#footnote-1)^** | **Number of reporting households (total n participating in projects within study villages = 329)** | **Mean importance rating** |
| --- | --- | --- | --- |
| **Economic benefits** | | | |
| Increased income | VSLAs (100/154)   - Easy access to loans - Returns on investments made using VSLA loans - Interest payments   *“After sharing money from the VSL I bought fertiliser which I used in vegetable farming and I got a lot of money from that.”* (Kasungu household, MTE 2014) | 135 | 3.00 |
|  | CA (22/156)   - Sale of increased yields - Reduced land requirements decreases rent payments - Reduced labour requirements free up time to engage in alternative income-generating activities - Reduced expenditure on agricultural inputs   *“The people practising CA are even able to sell [excess] maize”* (Dedza household) |  |  |
|  | Livestock production (12/65)   - Livestock sales provide quick access to cash   *“Livestock is a source of income for us which improves the household condition”* (Nsanje household) |  |  |
|  | Seed multiplication (11/62)   - Improves access to seeds for growing ‘cash crops’ which can be sold for profit - Reduces expenditure on agricultural inputs   *“We can sell and use proceeds to buy salt, soap, clothes and other things”* (Dedza household, MTE 2014) |  |  |
| Improved business opportunities | VSLAs (7/154)   - Access to capital facilitates business investments   *“When I need money I go to the VSLA and borrow so I can buy screens, batteries, chargers and other things. With these things I can make a profit”* (Dedza household) | 19 | 3.00 |
|  | CA (9/156)   - Reduced labour frees up time to engage in business activities   *“We save money; we do other jobs instead of being on the farm. We do business with the saved time.”* (Nsanje household, MTE 2014) |  |  |
| Improved asset ownership | VSLAs (47/154)   - Loans, investment returns and interest payments finance asset purchases (including clothing, furniture, bicycles, radios, kitchen utensils, livestock, oxcarts, solar panels, batteries, building materials, property) | 48 | 2.96 |
| **Food security benefits** | | | |
| Enhanced crop yields | CA, irrigation, seed multiplication (122/156, 25/35, 4/62, respectively)   - Activities improve agricultural productivity compared with traditional farming options   *“We get much more food through practising CA”* (Nsanje household) | 149 | 3.00 |
|  | Livestock production and forestry (13/65, 3/202, respectively)   - Manure and *Faidherbia Albida* trees — natural fertilisers — enhance soil quality and productivity   *“Some thorny trees…fertilise the soil. When leaves fall down they are left to decompose and it makes manure”* (Nsanje household) |  |  |
|  | VSLA (13/154)   - Increased and improved farm inputs (e.g. fertiliser) purchased using loans, investment returns and interest payments, improving productivity |  |  |
| Year-round harvesting | Irrigation (16/35)   - Access to water throughout the year allows for multiple harvests   *“Because of irrigation we have food up to the end of March [from first harvest in April] when previously we only had it until July”* (Nsanje household) | 44 | 3.00 |
|  | Seed multiplication schemes (28/62)   - Different crops and crop varieties reach harvest at times spread through the year, reducing dependence on staple crops (maize, sorghum, cassava) which are harvested only once |  |  |
| Improved food purchasing power | VSLA (27/154)   - Food for the household purchased using loans, investment returns and interest payments, improving productivity   *“VSL helps us buy more food”* (Kasungu household) | 27 | 3.00 |
| More nutritious diet | Malnutrition training (8/15)   - Households have improved dietary knowledge   *“[Because of the training] our children are healthier”* (Dedza household) | 18 | 3.00 |
|  | Seed multiplication (6/62)   - Dietary variety is provided by newly available food crops   *“New seeds have improved our diet”* (Kasungu household) |  |  |
| **Other development benefits** | | | |
| Improved firewood access | Forestry and improved cookstoves (14/202, 4/21, respectively)   - Regeneration and conservation of woodlands and boundary planting   *“In the past we were spending about four hours in the mountain fetching firewood and now we only spend about two hours*” (Dedza household, MTE 2014) | 18 | 3.00 |
| Better education for children | VSLA (6/154)   - Income benefits mean households can afford secondary school fees and uniforms (compulsory for school attendance) due to loans, investment returns and interest payments   *“I practise CA on cowpeas and pigeon peas. I sell the crops and send my children to secondary school from the profits”* (Dedza household) | 7 | 3.00 |
| Improved health | Improved cookstoves (4/21)   - Reduced incidences of smoke-related disease | 5 | 3.00 |

1. Sometimes, households attributed single benefits to multiple activities. Therefore, the combined numbers of households attributing a benefit to different activities may surpass total numbers of households who reported particular benefits. Only the main activities to which benefits were attributed are reported here. Hence, the combined numbers of households attributing a benefit to different activities may also be less than the total numbers of households who reported particular benefits. [↑](#footnote-ref-1)
