# Supplementary material for: Investigating Climate Compatible Development Outcomes and their Implications for Distributive Justice: Evidence from Malawi
Source: Environ Manage. 2017 May 24;60(3):436–53. doi: 10.1007/s00267-017-0890-8 (PMC5544806; doi:10.1007/s00267-017-0890-8)
Supplement: Supplementary file 4 — Supplementary Appendix D [file 267_2017_890_MOESM4_ESM.docx]

**Appendix D: Adaptation benefits resulting from ECRP, as reported by participating households in study villages**

| **Benefit** | **Main activities attributed to**  **(fractions denote households attributing a benefit to a particular activity relative to those who participated in the activity)^[[1]](#footnote-1)^** | **Number of reporting households (total n participating in projects within study villages = 329)** | **Mean importance rating** |
| --- | --- | --- | --- |
| Reduced vulnerability to dry spells due to improved soil moisture and quality | CA (73/156)   - Soil coverage and minimum soil disturbance help retain soil moisture   *“When rainfall has happened there is prolonged moisture due to the maize stalks”* (Dedza household) | 81 | 2.97 |
| Houses, assets and farmland protected from heavy rainfall and flooding | Forestry (27/202)   - Trees and vetiver grasses act as natural flood barriers   *“We plant trees and grasses along the rivers because they can be strong against the force of flood waters. We started two or three years ago and it is working!”* (Nsanje household) | 32 | 2.95 |
| Houses, assets and farmland protected from heavy winds | Forestry (16/202)   - Trees act as natural wind breaks   *“We have protection from heavy winds and now no problems arise”* (Kasungu household) | 16 | 3.00 |
| Ability to grow food throughout the year increases households’ abilities to deal with individual climate shocks | Seed multiplication (8/62)   - Access to crops with different maturity rates and harvest times lessens the impact of individual instances of flooding or drought - Access to crops (e.g. sweet potato, different maize varieties) considered more robust against climate shocks than staple food crops (e.g. traditional maize varieties)   *“When wind and rains strike it affects maize. But now I grow legumes as well which I sell and then buy maize for my family”* (Dedza household) | 8 | 3.00 |
| Access to emergency finance enables responses to the consequences of climate shocks | VSLA (33/154)   - Easy access to loans helps households repair damage caused   *“We used VSL money to buy thatching grass after the wind carried away the roof of our house”* (Nsanje household) | 34 | 3.00 |

1. Sometimes, households attributed single benefits to multiple activities. Therefore, the combined numbers of households attributing a benefit to different activities may surpass total numbers of households who reported particular benefits. Only the main activities to which benefits were attributed are reported here. Hence, the combined numbers of households attributing a benefit to different activities may also be less than the total numbers of households who reported particular benefits. [↑](#footnote-ref-1)
