# Supplementary material for: Investigating Climate Compatible Development Outcomes and their Implications for Distributive Justice: Evidence from Malawi
Source: Environ Manage. 2017 May 24;60(3):436–53. doi: 10.1007/s00267-017-0890-8 (PMC5544806; doi:10.1007/s00267-017-0890-8)
Supplement: Supplementary file 5 — Supplementary Appendix E [file 267_2017_890_MOESM5_ESM.docx]

**Appendix E: Issues which hinder the translation of project activities into CCD benefits**

| **Issue** | **Description** | **Reported impact(s)** | **Reported by** |
| --- | --- | --- | --- |
| **Agricultural activities** | | | |
| Negative perceptions of CA | - Traditional agricultural practises involve farmers digging the soil - Households are teased and abused by other villagers for participating in CA, which requires minimum soil tillage   *“Eight people have dropped out because when we were carrying stalks people were teasing us, saying ‘you are mad, why are you doing this?’”* (Dedza household) | - CA dis-adoption - Only small areas of land committed to CA | 9 households, 1 NGO employee |
| Delayed CA benefits | - Organic soil cover nutrients are fully absorbed into soils only after two to three years, leading to delayed development and adaptation benefits   *“You see soil fertility benefits after two or three years. So if I am a farmer who is quite sceptical and I am forced to adopt CA and after a year I don’t see results then it is very probable that I quit”* (NGO employee) |  | 2 households, 2 NGO employees |
| Poor fertiliser access | - Application of synthetic fertilisers can help offset delayed CA benefits but household access is poor   *“Until [CA benefits emerge] fertiliser application needs to be intensive but we don’t have fertiliser so we experience poor harvests”* (Kasungu household) | - Poor harvests - CA dis-adoption | 7 households, 1 NGO employee |
| Pest attacks | - Insects and weeds damage crops and organic soil cover   *“It looks like we are not practising CA because there are no stalks [due to termites]”* (Nsanje household) | - Poor harvests - CA benefits lost | 9 households |
| Co-existence with livestock and other animals | - Goats and baboons eat and damage crops and organic soil cover   *“One of the pillars of CA is to leave crop residues on the field. But this must be compatible with the presence of livestock. And the co-existence is not easy.”* (NGO employee) |  | 7 households, 3 NGO employees |
| Expense of irrigation scheme upkeep | - Households cannot afford to replace irrigation infrastructure when it breaks down   *“Small-scale irrigation, when it breaks down we don’t know if they will have enough money to make sure that it starts working”* (Donor employee) | - Irrigation benefits lost | 6 households, 1 donor employee |
| Seed replacement | - Households cannot afford to replace seeds required for multiplication schemes   *“For those hybrid seeds you need to buy new seeds; that will be a problem”* (NGO employee) | - Seed multiplication benefits lost | 1 NGO employee |
| Poor market access | - Households — especially residents of remote villages — have inadequate access to suitable markets for selling cash crops   *“Vendors come and will only buy for very low prices. But we have no other options because there is no nearby place to sell”* (Kasungu household) | - Agricultural activity benefits reduced or lost | 2 households, 1 NGO employee |
| Extreme weather events | - Droughts and severe dry spells compromise benefits of agricultural activities - Heavy rains destroy crops and organic soil cover and undermine CA soil fertility gains - Heavy rain can lead to waterlogging when CA is practised   *“Even under CA, soil moisture is not enough”* (Nsanje household)  *“It can be very difficult when the rains come as the crops and mulch are washed away and the goodness in the soil is lost”* (Nsanje household)  *“Those that mulched their fields have experienced waterlogging”* (NGO employee)  *“*(Seed) p*ass-on is difficult when weather conditions are bad”* (Nsanje household) | - Agricultural activity benefits reduced or list - Seed pass-on compromised - Poor harvests - CA dis-adoption | 26 households, 2 NGO employees |
| **Livestock production** | | | |
| Prioritisation of short-term benefits | - Households eat or sell livestock shortly after pass-on to access food and income quickly or in response to climate and development shocks   *“Once people pass-on, they sell goats to make short-term cash”* (Kasungu household) | - Sustainable livestock production benefits (e.g. access to manure, goats milk) lost | 14 households |
| **Forestry** | | | |
| Communal, non-immediate benefits | - Participating and non-participating households benefit similarly from afforestation. Households are disillusioned about participating in afforestation, which does not yield immediate benefits, for ‘free’. They would like to receive additional, immediate benefits in return for their labour   *“[Participants] don’t work hard as it is for the group not individuals”* (Nsanje household) | - Limited participation in forestry activities - forestrybenefits reduced or foregone | 3 households, 1 NGO employee |
| Extreme weather events | - Dry spells and drought mean tree seedlings do not receive enough water - Heavy rains and floods damage and destroy trees   *“The trees were washed away by flooding”* (Nsanje household) | - Forestry benefits reduced or foregone | 8 households |
| **VSLA** | | | |
| Drop-outs | - VSLA members struggle to pay back loans and are forced to withdraw from groups.   *“Only three quarters of our group can pay back on time”* (Kasungu household) | - Reduced availability of loans | 32 households |
| Challenges for doing business | - Financial poverty translates into limited markets for new businesses - Low education levels limit innovation which is required for business success   *“People just copy each other’s business ideas, which drives prices down”* (Nsanje household) | - Business profits limited | 4 households |
| **Low-carbon technologies** | | | |
| Limitations of market-based approaches | - Financial poverty in ECRP target villages makes it difficult for households to afford products - Unsensitised households in non-ECRP target villages are unaware of products   *“Targeting the bottom 10% is not ideal. They have just enough to pay for phones…they have breathing room. But I think you will observe when you go to communities that those who buy the (solar) lights are not the poorest”* (NGO employee) | - Low affordability and lack of awareness reduces markets for solar products and cookstoves - Few have capital required to become solar entrepreneurs | 4 NGO employees |
| Opportunity costs of improved cookstove production | - Other livelihood options are more profitable than cookstove production - DISCOVER pledged to top-up income from cookstove sales with money obtained from carbon credit sales, but this has yet to materialise   *“Returns are not high compared with other options to make money [in Dedza], like rice cultivation or maize production…Producers sell cookstoves for 500MKW and should receive another 500MKW from carbon finance. [But] they have not received the carbon money…if they received this it would help a lot”* (NGO employee) | - Stove production eschewed in favour of other livelihood activities | 3 NGO employees |
| Cheaper solar products available | - Cheaper solar products than those sold under ECRP are available - Poor quality of alternative products deters investments in solar   *“There are cheaper Chinese products available”* (NGO employee) | - Products unaffordable - Solar entrepreneurship too capital intensive | 4 NGO employees |
| **All activities** | | | |
| Patchy extension worker services | - Extension worker performance across Malawi is patchy. Reduced training and policing of project activities could create problems in villages without sufficient support once ECRP comes to an end   *“Extension Workers have not come to this village since 2013”* (Kasungu household)  *“Pass-on [of livestock and seeds] will not continue [in villages without extension support] unless local leadership apply pressure…people will just think ‘why should I pass on’?”* (NGO employee) | - Households receive insufficient technical advice - Reduced incentives to spread project resources within villages | 12 households, 2 NGO employees |
