# Supplementary material for: Investigating Climate Compatible Development Outcomes and their Implications for Distributive Justice: Evidence from Malawi
Source: Environ Manage. 2017 May 24;60(3):436–53. doi: 10.1007/s00267-017-0890-8 (PMC5544806; doi:10.1007/s00267-017-0890-8)
Supplement: Supplementary file 6 — Supplementary Appendix F [file 267_2017_890_MOESM6_ESM.docx]

**Appendix F: Side-effects resulting from ECRP, as reported by households in study villages**

| **Cost** | **Description** | **Households experienced by** | **Mean importance rating** |
| --- | --- | --- | --- |
| Increased inequality within villages | Uneven opportunities to participate in project activities mean some people within villages are able to improve their lives while others are not.  *“They (participating households) get wealthier while we are just left behind”* (Nsanje household) | 16 | 2.94 |
| Loss of money and assets | - Households lose money when VSLA members fail to pay back loans   *“Many people are not able to give back loans…which makes others suffer”* (Nsanje household)   - Households who fail to pay back loans to VSLAs, and their family, have property confiscated   *“Debt collectors took two goats from me while my son paid his debt but I never got them back”* (Kasungu household)   - Households are asked to spend time and resources building corrals to show they are ‘capable’ of keeping livestock. Some have taken loans to afford construction materials. However, they have not all received livestock from DISCOVER   *“There have been cases whereby we say no, you have the corral but you are not fit”* (NGO employee) | 9 | 2.88 |
| Increased crime within villages | Increased resource wealth through project activities has led to greater instances of theft (e.g. of livestock and crops grown under irrigation) within villages.  *“People steal the goats and this affects the progress of the project” (*Nsanje household) | 3 | 3.00 |
| Reduced crop yields | Under conditions of heavy rainfall, CA leads to waterlogged fields and reduced crop yields.  *“Waterlogging is bad in some fields [in which CA is practiced], especially when the topography is not suitable”* (Nsanje household) | 3 | 3.00 |
| Opportunities to carry out important livelihood activities foregone | - Households in one Dedza village have participated in afforestation but poor access to water has constrained tree growth and prevented benefits   *“There are no actual benefits. We are unsure what benefits will come”* (Dedza household) | 5 | 3.00 |
