# Supplementary material for: Investigating Climate Compatible Development Outcomes and their Implications for Distributive Justice: Evidence from Malawi
Source: Environ Manage. 2017 May 24;60(3):436–53. doi: 10.1007/s00267-017-0890-8 (PMC5544806; doi:10.1007/s00267-017-0890-8)
Supplement: Supplementary file 7 — Supplementary Appendix G [file 267_2017_890_MOESM7_ESM.docx]

**Appendix G: Auxiliary benefits experienced by professional stakeholders at supralocal governance levels**

| **Benefit** | **Description** | **Stakeholder experienced by** | **Mean importance rating** |
| --- | --- | --- | --- |
| Increased organisational capacity | ECRP training has enhanced District Government employees’ expertise around disaster risk reduction, climate change and rural development.  *“We have been trained in different types of activities…Now we have the knowledge and that knowledge will still be there for long”* (District Government Employee) | 10 District Government Employees | 2.90 |
| Increased organisational innovation | Working as part of Consortia has provided implementing NGOs with opportunities to engage with new climate change and development approaches; presenting opportunities for learning and innovation.  *“Certain aspects of the project were not done previously but we can learn from other implementing partners coming together. We are doing new things and testing out new ideas.”* (NGO Employee) | 13 NGO Employees | 3.00 |
| Improved reputation | Involvement in Malawi’s flagship climate-development programme has brought NGOs positive publicity. Perceived ECRP success has enhanced host governments’ perceptions of donor agencies.  *“When the projects are discussed in the Commons and the House of Lords, they say ‘DfID is really improving peoples’ lives’. [ECRP] helps convince Government that DfID is improving peoples’ lives”* (Donor Employee)  *“[ECRP] gave us some visibility, more recognition among stakeholders around the country”* (NGO Employee) | 8 NGO Employees, 1 Donor Employee | 2.71 |
| Access to finance and material resources | ECRP resources (finance, fuel, protective clothing for field workers) allow NGOs and Local Government to continue their core operations.  *“DISCOVER is the largest contract [redacted NGO name] has ever had, not just in Malawi but worldwide”* (NGO Employee)  *“It means the district has got money in terms of implementing these activities, so as part of the government we are relieved”* (District Government Employee) | 9 NGO Employees,  3 District Government Employees | 2.92 |
| Enhanced lobbying influence | Lobbying and developing policy positions as part of consortia, NGOs have greater influence over public decision-making.  *“We are reaching a larger audience. DISCOVER has developed materials and literature…This allows us [NGOs] to contribute to wider debates and discussions”* (NGO Employee) | 2 NGO Employees | 2.50 |
| District government cohesion and relationship building | Involvement in ECRP has improved co-ordination within District Government: Departments have better relationships with one another and development is considered holistically.  *“Local Government is now more joined up…. Now we write integrated proposals to NGOs which combine different aspects of development”* (District Government Employee) | District Government Employee | 3.00 |
